# What Are the Mechanisms Underpinning Intergenerational Transmission of Violence Perpetration? A Realist Review

**DOI:** 10.1177/15248380251361468

**Published:** 2025-09-01

**Authors:** Franziska Meinck, Mengyao Lu, Deeksha Suresh, Melis Cetin, Lakshmi Neelakantan, Chad Hemady, Hannabeth Franchino-Olsen, Nataly Woollett, GJ Melendez-Torres, Andrea Gonzalez, Nicola Christofides

**Affiliations:** 1 University of Edinburgh, UK; 2 University of the Witwatersrand, Johannesburg, South Africa; 3 North-West University, Vanderbijlpark, South Africa; 4 University of Edinburgh, UK; 5 University of Pittsburgh, PA, USA; 6 Bogazici University, Istanbul, Turkey; 7 University of Melbourne, VIC, Australia; 8 Youth Endowment Fund, London, UK; 9 The Ohio State University, Columbus, USA; 10 University of Johannesburg, South Africa; 11 University of Exeter, UK; 12 McMaster University, Hamilton, ON, Canada

**Keywords:** intergenerational, violence, child abuse, intimate partner violence, perpetration

## Abstract

Successive generations are more likely to carry out acts of violence in households where an individual has either perpetrated or been subjected to violence. While research to date has mostly concentrated on direct associations between violence experience or perpetration in generation 1 and violence perpetration in generation 2, there is limited evidence regarding the underlying mechanisms of the transmission of intergenerational violence perpetration. We conducted a realist review to adjudicate theories of the underlying mechanisms of intergenerational violence perpetration. Following Realist and Meta-Review Evidence Synthesis: Evolving Standards, we searched six databases across three phases considering all study designs. We identified 28 studies that were analyzed thematically. Included studies focused on perpetration of intimate partner violence, child abuse and neglect, youth violence, and violent crime. We identified five underlying mechanisms of intergenerational violence perpetration: (a) normalization of violence and harmful gender norms, (b) modeling and imitation, (c) emotion dysregulation, (d) high vulnerability, and (e) impaired relationships. These mechanisms operate and unfold differently across contexts where violence is experienced or witnessed. Our realist review highlights how violence perpetration may be transmitted across generations. We propose that interventions focused on norms and attitudes, mental health, social welfare, and parent–child relationships may be useful in preventing violence perpetration across generations.

## Introduction

Exposure to violence has been consistently associated with detrimental outcomes across the life course. Individuals exposed to violence are at higher risk of injury, infectious diseases, and mental health problems in later years (Hillis et al., 2017). In studying the effects of exposure to violence, there has been a consistent and long-standing interest in the so-called “cycle of violence” or the intergenerational transmission/continuity of violence and the question as to why people become perpetrators of violence. The notion of violence transmission is essentially concerned with whether an adult’s experience of violence across their life course increases the risk that they will then perpetrate violence toward their offspring, or that their offspring will also perpetrate or experience violence (Madigan et al., 2019). The violence can occur within and outside of the family. Transmission of violence victimization occurs when a person in generation 1 (G1) experiences violence and their offspring in generation 2 (G2) also experience violence. Transmission of violence perpetration occurs when generation 1 (G1) experiences or perpetrates violence and their offspring (G2) then perpetrate violence. Researchers distinguish between two types of transmission: homotypical transmission, where the same type of violence is perpetuated across generations, and heterotypical transmission, where a different type of violence is experienced/perpetrated in G1 compared to G2. Homotypical transmission is, for example, the use of violence against a child in both G1 and G2, while heterotypical transmission is child maltreatment experience in G1 and IPV perpetration in G2. This distinction is important because different types of violence have non-overlapping risk and protective factors and are associated with different outcomes across the life course, therefore making a differential approach to violence transmission necessary to tailor prevention strategies (Langevin et al., 2023).

Generally, research on intergenerational transmission of violence perpetration has focused exclusively on family violence or violent delinquency. Evidence for intergenerational transmission is available in multiple systematic reviews: for child abuse perpetration (G1) and subsequent child abuse perpetration (G2); intimate partner violence experience (IPV) (G1) and subsequent IPV perpetration (G2); and IPV perpetration (G1) and subsequent child abuse perpetration (G2), all with small to modest effect sizes (Assink et al., 2018; Ertem et al., 2000; Gerino et al., 2018; Greene et al., 2020; Langevin et al., 2019; Madigan et al., 2019; Savage et al., 2019; Schofield et al., 2013; Stith et al., 2000; Thornberry et al., 2012). A key finding in many of these reviews is that there are significant methodological limitations in primary studies examining the transmission of violence. For instance, many studies examine child maltreatment or IPV but there is little information about the subtypes of violence included, nor their individual associations with intergenerational transmission (Langevin et al., 2023).

While much literature does support that transmission of intergenerational violence perpetration occurs, there are some studies that dispute a link between violence in G1 and perpetration of violence in G2. A recent umbrella synthesis of 19 meta-analyses examining child maltreatment antecedents and interventions has confirmed the persistent observation that being exposed to violence does increase violence perpetration in subsequent generations (van IJzendoorn et al., 2020), and the authors established a 65% chance that parents who have experienced maltreatment will go on to maltreat their own children. The current body of evidence focuses almost exclusively on IPV, physical and emotional child abuse, and violent offending, and less is known about the transmission of other types of violence (e.g., peer violence, sexual abuse, sexual exploitation).

Multiple theoretical frameworks on the intergenerational transmission of violence perpetration exist. These have different lenses depending on the subject area they stem from; for example, literature on the intergenerational transmission of criminal behaviors often focuses on criminogenic environments (Farrington, 2011) while research focused on the intergenerational transmission of IPV utilizes predominantly social learning theory (Bandura, 1977), and that on the intergenerational transmission of child maltreatment often follows a socio-ecological framework (Bronfenbrenner, 1979). An overview of the most common theoretical frameworks on intergenerational violence perpetration, which have considerable overlap with one another, can be found in Supplement 1.

While several existing theoretical frameworks focus on the role of context, that is, criminogenic environments or risk factor frameworks (Farrington, 2011), few empirical studies have adjudicated these theories to determine why certain families or circumstances are more likely to experience intergenerational cycles of violence than others. Therefore, it is important to explore contexts and mechanisms through which violence could be transmitted to affect violence perpetration outcomes, both inside and outside of the home. Moreover, despite the important role of culture in the definitions and occurrence of violence, there is a paucity of research examining societal and cultural influences on why and how violence transmission takes place (Kim, 2012). Realist perspectives enable us to address this important gap in the evidence due to their focus on understanding what happens to whom under which circumstances and why (Pawson & Manzano-Santaella, 2012) and allow us to assess the relative merits and contextual contingencies relevant to each theory.

Identifying the root causes and pathways through which violence perpetuates across generations can significantly improve interventions to disrupt these cycles of violence. Without interruption, violence continues, and policies and programs, which largely continue to be siloed, fail to address the overlap between different types of violence for example, child maltreatment and IPV. This means that the most vulnerable are slipping through the net. The consequences of violence transmission are significant at both the individual and societal level: increased public health burden of mental health, injuries and chronic health conditions (Widom, 2012) associated with significant economic costs putting a strain on already limited resources and impairment of children’s development (Herbert et al., 2025; Pereznieto et al., 2014), hindering their ability to contribute as productive members of society are coupled with an undermining of community safety and social cohesion leading to a distrust in institutions (Randau, 2021). This realist review aimed to provide evidence on the mechanisms by which violence experience in one generation (G1) can increase the risk of perpetration of violence in the second generation (G2). This review aimed to answer the following research question: How, why, and under what conditions does experience or perpetration of violence in G1 increase the risk of violence perpetration in G2?

## Methods

Realist reviews operate on the premise that there are one or more causal processes for any observed outcome that become active in certain contexts (Wong, 2018). In order to infer a causal outcome (0) between two events, one needs to understand the underlying mechanism (M) that connects them to the context (C) in which this relationship occurs (Pawson et al., 2005). Realist reviews are theory-driven and involve gathering and categorizing key theories and tracing evidence to confirm or refute them, thereby ensuring that the focus remains on mechanisms that link contexts and outcomes. Realist approaches also do not prioritize qualitative or quantitative evidence and see the value in combining both types of evidence during the review process (Kastner et al., 2012). This realist review followed a procedure based on the five steps laid out by Pawson et al. (2005), which was modified to allow for focus on non-intervention focused studies and which did not include dissemination and evaluation to stakeholders other than in academia.

A review protocol was developed and registered on PROSPERO (2021:CRD42021267725). The scope of this protocol was to investigate intergenerational transmission of violence victimization and perpetration, to present results on both in a single review. The nature and focus of the evidence resulted in two separate articles, one on intergenerational transmission of violence perpetration and one on violence victimization (Lu et al., under review).

### Search for Evidence

We employed a three-stage search strategy for this review. This is because several recent reviews have been conducted on intergenerational transmission of violence victimization and perpetration, most commonly the overall effect of exposure to child maltreatment and later parenting and IPV perpetration (Greene et al., 2020; Langevin et al., 2019; Madigan et al., 2019; Savage et al., 2019). This means a new extensive primary search focusing on quantitative research was not likely to help fulfil the aims of this review.

We searched the following electronic databases between January 1990 until December 2021: EMBASE, Global Health, PsycINFO, Sociological Abstracts, Cochrane Library, ProQuest Dissertations and Theses and also checked reference lists of included studies, lists of studies excluded from other reviews and meta-analyses, searched forward and back citations, contacted stakeholders in the field and searched grey literature, namely the Sexual Violence Research Initiative and International Society for the Prevention of Child Abuse and Neglect conference abstract summaries and World Health Organization and UNICEF websites (see Table 1 for search stages and Supplement 2, 3, and 4 for searches).

**Table 1.** Search Stages.

Search Stage	Search Stage 1	Search Stage 2	Search Stage 3
Search strategy	See Supplement 1 Up to May 31, 2021	See Supplement 2 Up to November 1, 2021	See Supplement 3 Up to December 31, 2021
Types of studies	Systematic reviews and meta-analyses on intergenerational violence transmission	Qualitative studies of types of violence identified in Stage 1	In-depth search for qualitative and quantitative studies of intergenerational transmission of violence types other than those identified in Stages 1 and 2
Rationale for search	Many systematic reviews and meta-analyses on whether transmission happens and size of effect, this search focused on the explanation of underlying mechanisms for violence transmission as stated in systematic reviews/meta-analyses	Previous search focused on systematic reviews and meta-analyses, which included almost exclusively quantitative studies, hence the need to focus on qualitative studies to understand underlying mechanisms of the types of violence transmission investigated in the included reviews	Specific search for types of violence transmission of which other included studies had not provided evidence, particularly extra-familial types of violence, e.g., bullying
Inclusion	(a) Systematic reviews or meta-analyses; (b) violence experienced/perpetrated by G1 and perpetrated by G2; (c) mechanisms of intergenerational violence transmission	(a) Qualitative studies on intergenerational transmission of IPV, child maltreatment, child abuse and crime involvement	(a) Any study design; (b) intergenerational transmission of bullying, sexual exploitation, gang violence, youth violence, community violence
Exclusion	(a) Non-empirical work; (b) studies that were not systematic reviews; (c) violence experienced/perpetrated by only one generation	(a) All other violence types; (b) all other study designs, (c) violence experienced/perpetrated only by one generation	(a) Non-empirical work; (b) studies focused on child abuse and neglect, child maltreatment, IPV and crime involvement; (c) violence experienced/perpetrated only by one generation

### Study Selection and Data Extraction

Two reviewers screened titles and abstracts and extracted data. Conflicts were resolved in discussion with a third reviewer. In considering relevant studies, we included information not only in the results or findings sections but also in other sections such as the discussion explaining the implications of findings or the introduction, which provides descriptions of theories. Searches were limited to the English language, and all countries and settings were considered.

We screened all titles and abstracts using the following inclusion criteria: (a) studies which focused on intergenerational transmission of violence perpetration; (b) described or contained information that explained mechanism of intergenerational violence transmission such as mediator analysis; (c) violence experienced by G1 was broadly defined as including all types of interpersonal violence that is, peer violence, child abuse and neglect, IPV, violent criminal behavior, community violence; (d) and where violence was witnessed or experienced in G1 and then subsequently perpetrated by G2, (e) violence in G2 was also broadly defined and included all types of interpersonal violence. Except for Stage 1, where we specifically searched for published reviews, all types of study designs (e.g., qualitative, quantitative, mixed methods) were considered in the searches for Stages 2 and 3. We excluded opinion pieces, editorials, non-expert commentary, and letters. Studies were selected based on the contribution they made to the problem theory of mechanisms underlying intergenerational transmission of violence perpetration.

Four reviewers extracted general study characteristics, context, mechanisms, and outcomes, as well as the richness of data and supporting evidence for mechanisms. We prioritized studies based on their relevance and rigor. Relevance focused on whether the study provided evidence in relation to the problem theories that were being tested, rigor on the robustness and credibility of the conclusions drawn by the original study authors (Wong et al., 2013).

### Synthesize Evidence and Draw Conclusions

We synthesized extracted data to interrogate theories on violence transmission to determine when transmission takes place, under what circumstances, for whom, and why to support and adjudicate existing theories. A structured coding framework was developed to systematically capture context (C), mechanism (M), and outcome (O) across various types of violence exposure examined in the included studies. Each reviewer was responsible for coding a subset of studies using the coding framework. Emerging CMO patterns were compared across studies and iteratively synthesized to develop overarching narrative explanations for intergenerational transmission of violence perpetration. The findings from the narrative synthesis are presented as a final problem theory in the form of multiple CMO configurations. Discrepancies were resolved through regular discussion with the full author team, and the coding framework was revised as necessary. Contradictory evidence was scrutinized to generate insights about contextual influences (Pawson et al., 2005). We reported findings in accordance with the Realist and Meta-Review Evidence Synthesis: Evolving Standards (RAMESES) publication standards (Wong et al., 2013).

## Results

**Figure 1.**
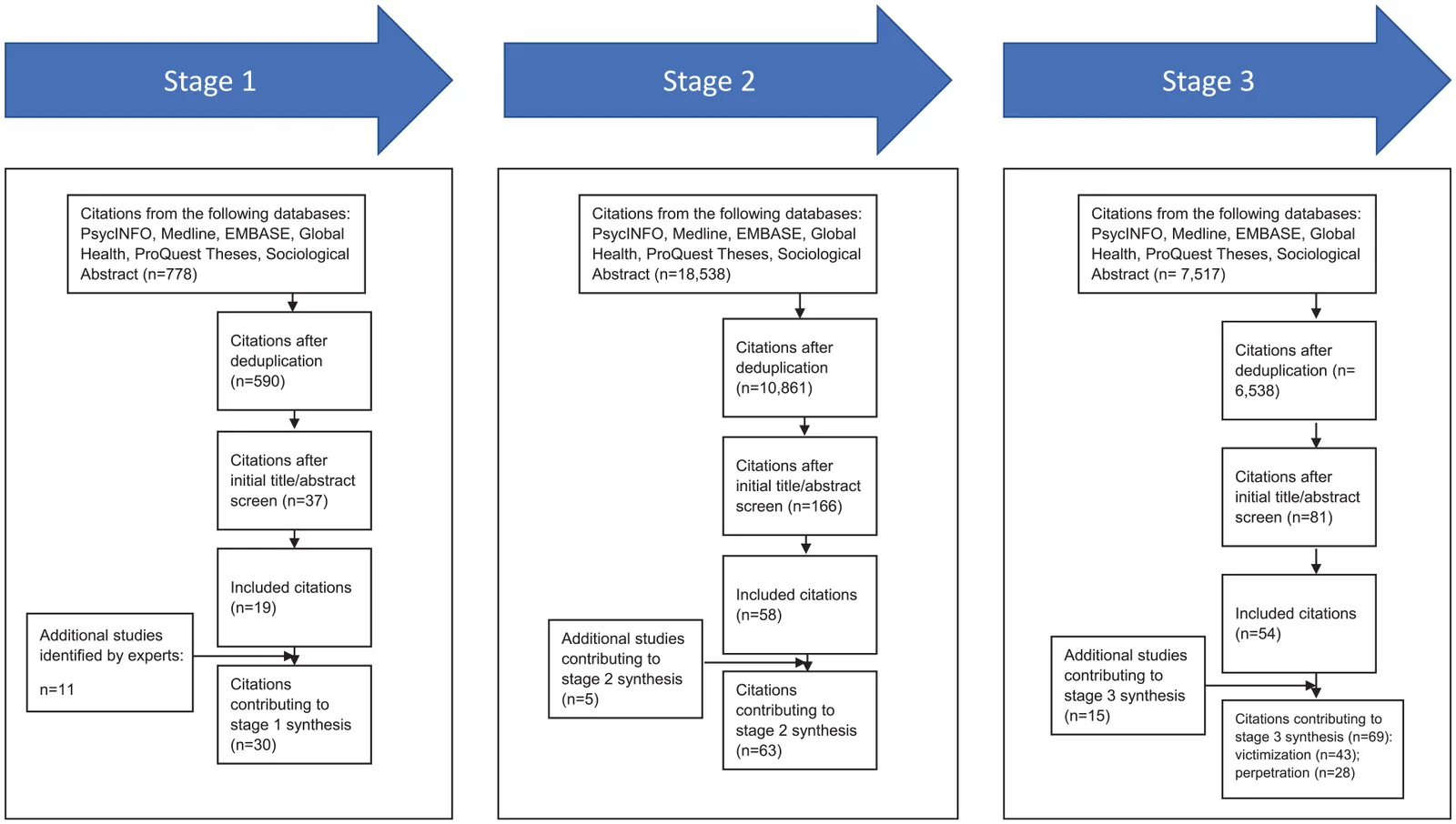
RAMESES flow diagram of search and screening processes and included studies (Wong et al., 2013).

In total, 28 studies on perpetration pathways were included via three search rounds (see RAMESES flowchart in Figure 1), 20 of which were primary studies (5 qualitative; 15 quantitative), and 8 were systematic reviews or meta-analyses. Victimization pathways are subject to another manuscript (Lu et al., under review). Included studies covered populations in North America (*n* = 10) (Augustyn et al., 2017; Bozzay et al., 2020; Chapple, 2003; Fite et al., 2008; Kim et al., 2009; Martinez, 2020; Narayan et al., 2013; Reyes et al., 2015; Smith et al., 2011; Thornberry et al., 2013), the Caribbean (*n* = 1) (Gage, 2016) , Asia (*n* = 3) (Choi et al., 2023; Li et al., 2021; Murshid & Murshid, 2018) and Europe (*n* = 6) (Adshead & Bluglass, 2001; Domoney & Trevillion, 2021; Frisell et al., 2011; Jaffee et al., 2013; Spapens & Moors, 2020; van Dijk et al., 2019), with seven being global reviews (Besemer et al., 2017; Goncy, 2020; Goncy et al., 2021; Greene et al., 2020; Kimber et al., 2018; Li et al., 2020; Stith et al., 2000) and one reviewing evidence from the United Kingdom and the United States (Schofield et al., 2013). Three studies used data from the Rochester Development Study (Sameroff et al., 1987; Augustyn et al., 2017; Smith et al., 2011; Thornberry et al., 2013).

Sixteen of the included studies focused only on homotypical continuity of violence perpetration. A smaller number focused on heterotypical continuity (*n* = 4).

Studies investigated homotypical continuity of IPV (*n* = 8) (Chapple, 2003; Fite et al., 2008; Gage, 2016; Goncy, 2020; Kim et al., 2009; Kimber et al., 2018; Martinez, 2020; Murshid & Murshid, 2018), violent criminal behaviors (*n* = 5) (Augustyn et al., 2017; Besemer et al., 2017; Frisell et al., 2011; Spapens & Moors, 2020; van Dijk et al., 2019), and child maltreatment (*n* = 3) (Greene et al., 2020; Schofield et al., 2013; Thornberry et al., 2013). Heterotypical continuity was investigated for parental IPV (G1, witnessed by G2) or physical abuse of G2 by G1 and youth violence perpetration (G2) ( Li et al., 2021); child maltreatment (G1) and IPV perpetration (G2) ( Li et al., 2020) child maltreatment (G1) and fictitious illness (G2) (Adshead & Bluglass, 2001); and child maltreatment by G1 against G2 and subsequent dating aggression (G2) (Goncy et al., 2021). Studies focused on both homo- and heterotypical transmission examined: parental family violence (witnessed by G2) or experience of child maltreatment (G2) and subsequent violent aggression against parents and peers (G2) (Bozzay et al., 2020); IPV in adulthood/experience of child maltreatment (G1) and dating aggression (G2) (Reyes et al., 2015); child maltreatment experience or IPV victimization in adulthood (G1) and subsequent violent parenting (G2) (Choi et al., 2023; Jaffee et al., 2013); and IPV of G1 (self-reported or witnessed by G2) in childhood or child maltreatment against G2 and subsequent IPV perpetration (G2) (Domoney & Trevillion, 2021; Narayan et al., 2013; Reyes et al., 2015; Smith et al., 2011; Stith et al., 2000).

**Table:** Critical Findings.

• This review identified five mechanisms that underly intergenerational transmission of violence perpetration: (a) normalization of violence and harmful gender norms; (b) modeling and imitation; (c) emotion dysregulation; (d) high vulnerability; and (e) impaired relationships • Evidence is predominantly available for the transmission of IPV, child maltreatment, and violent criminal behavior, and little is known about the different subtypes of violence nor the situational context of perpetration • Findings support existing theories on familial intergenerational violence transmission including power theory, social learning theory, stress process model, socio-ecologic systems theory, criminogenic environments, and attachment theory.

Of all included studies, four investigated intergenerational continuity of violence from mothers to offspring (Adshead & Bluglass, 2001; Choi et al., 2023; Jaffee et al., 2013; Narayan et al., 2013), two from fathers to offspring (Domoney & Trevillion, 2021; Murshid & Murshid, 2018), ten reported on each parent, respectively (Augustyn et al., 2017; Besemer et al., 2017; Fite et al., 2008; Gage, 2016; Goncy, 2020; Goncy et al., 2021, p. 202; Greene et al., 2020; Kim et al., 2009; Kimber et al., 2018; Martinez, 2020) and the rest did not distinguish between parents. For most studies, there was no information as to who the perpetrator or victim of the violence in G1 was, and who had inflicted child maltreatment against G2. Studies focused on IPV, and child maltreatment typically focused on physical and emotional abuse, and few focused on economic or sexual abuse and neglect; none provided the situational context in which the perpetration occurred (e.g., perceived misbehaviour, aggression between parents and child gets involved). No studies focused on intergenerational transmission of peer violence, community violence, or sexual exploitation.

Nine primary studies included data from one generation only (Chapple, 2003; Choi et al., 2023; Domoney & Trevillion, 2021; Gage, 2016; S. D. Li et al., 2021; Martinez, 2020; Murshid & Murshid, 2018; Reyes et al., 2015; Thornberry et al., 2013), eight from two generations (Augustyn et al., 2017; Bozzay et al., 2020; Fite et al., 2008; Jaffee et al., 2013; Kim et al., 2009; Narayan et al., 2013; Smith et al., 2011; van Dijk et al., 2019), and two qualitative studies and one quantitative study from three generations (Adshead & Bluglass, 2001; Frisell et al., 2011; Spapens & Moors, 2020).

Findings are presented as a final problem theory in the form of context–mechanism–outcome (CMOs) configurations below. The review identified five CMOs: (a) normalization of violence and harmful gender norms; (b) modeling and imitation; (c) emotion dysregulation; (d) high vulnerability; and (e) parenting and attachment. Please see Supplement 5 for included studies and their overall findings.

### Synthesis of Evidence

#### CMO 1: Normalization of Violence and Harmful Gender Norms

**Figure 2.**
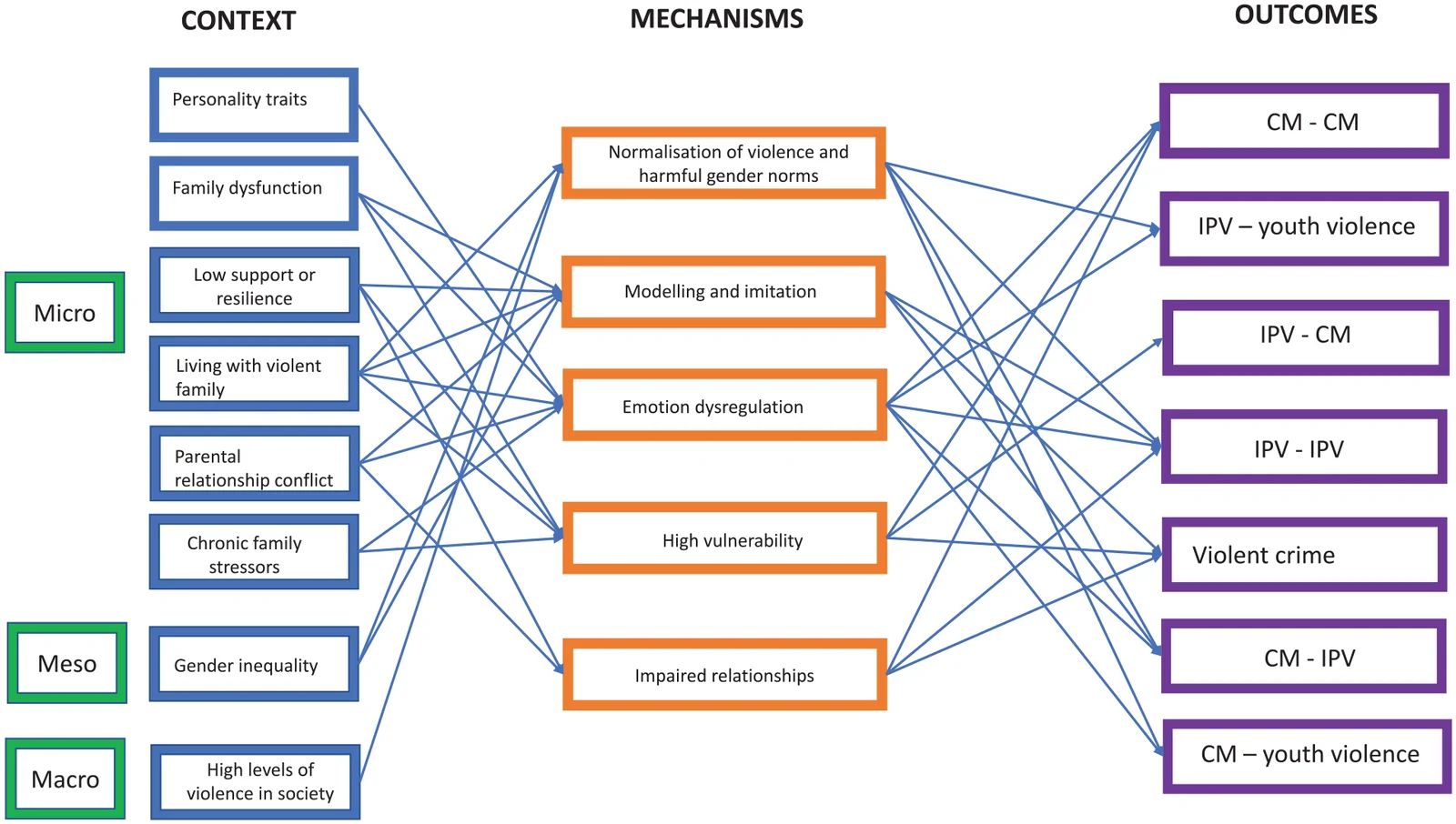
Final CMO configurations for the underlying mechanisms of the transmission of intergenerational violence perpetration.

***If*** the parents were violent in the presence of or toward their child, in contexts characterized by high levels of violence and gender inequality, (C) (G1), ***then*** the child normalizes and justifies violence and adopts harmful gender norms (G2; M), which might result in **violence perpetration** in both adolescence and adulthood (O) (G2) (Figure 2).

Seven included studies supported this CMO focused on the cognitive phenomenon of “normalization,” where one’s schema about violence and the acceptability of it is altered by the broader context in which one lives (Domoney & Trevillion, 2021; Gage, 2016; Kimber et al., 2018; Li et al., 2021; Martinez, 2020; Reyes et al., 2015; Stith et al., 2000). Of these, three studies focused on homotypical continuity of IPV (Gage, 2016; Kimber et al., 2018; Martinez, 2020), and three studies focused on heterotypical continuity of violence in the following combinations: IPV (G1)/child maltreatment perpetration (G1) to youth violence (G2)(Li et al., 2021), IPV (G1)/Child maltreatment perpetration (G1) to IPV perpetration (G2) (Domoney & Trevillion, 2021; Reyes et al., 2015; Stith et al., 2000). Normalization is best explained by power theory, which posits that normative attitudes and social acceptance of violence, gender inequality, and family conflict intersect with each other at the structural level to increase tensions within a family. Economic hardships, power imbalances between partners and family members, and high levels of stress increase risk for use of violence, and witnessing or experiencing violence results in children learning that violence is acceptable to resolve conflicts (Straus et al., 1980).

A cross-sectional survey examined exposure to parental spousal violence and physical, psychological and sexual dating violence perpetration with 342 high school students in Port-au-Prince, Haiti, which has seen frequent coups d’états and civil unrest as well as street violence and domestic violence amongst other human rights violations, in addition to high levels of poverty, inequality and rapid urbanization (Gage, 2016). The authors state that violence is transmitted through the belief system where perceived peer tolerance to violence was a stronger factor associated with IPV perpetration than one’s own tolerance of violence. Therefore, children who observe violence in their families of origin and think their peers endorse violence may grow up to believe that violence is an appropriate way to resolve conflict in dating relationships (Gage, 2016). This is in support of both cultural cognitive theory, which posits that children who grow up in a culture that endorses violence, and experience parental violence, then also use violence to resolve conflict (Nisbett, 2003) and power theory, which sees the roots of violence transmission in both culture and family structure (Straus et al., 1980). Similarly, in a qualitative study with six Latino young adults who had witnessed domestic violence as children, Martinez (2020) found that witnessing domestic violence normalizes the use of violence in relationships and distorts views of what healthy relationships look like. As such, harmful gender norms were adopted, and violence was inflicted on intimate partners (Martinez, 2020).

In their longitudinal study of 1,100 adolescents in a Chinese metropolis, Li et al. (2021) found that children who witnessed family violence were more likely to engage with violent peers, which increased their risk for engaging in adolescent violence. These pathways were fully mediated by normative beliefs. The authors argue that children growing up in households characterized by violence would adopt behaviors carried out by their parents to solve conflicts; however, more impactful on adolescents’ use of violence is that those who experience violence frequently approve of it and associate with peers who carry the same normative beliefs and this is particularly true for boys (Li et al., 2021).

#### CMO 2: Modeling and Imitation

**If** parents engage in violence in the presence of their children (C) (G1), **then** their children have fewer chances to learn non-aggressive responses, and they may imitate their parents’ behavior (G2) (M), which can result in the perpetration of violence (O).

This CMO was supported by 12 included studies focused on the behavioral actualization of the schema where violence is acceptable (Augustyn et al., 2017; Fite et al., 2008; Gage, 2016; Goncy, 2020; Goncy et al., 2021; Kimber et al., 2018; Li et al., 2020; Murshid & Murshid, 2018; Smith et al., 2011; Spapens & Moors, 2020; Stith et al., 2000; van Dijk et al., 2019). Of these, ten focused on the homotypical continuity of violent criminal behavior (Augustyn et al., 2017; Spapens & Moors, 2020; van Dijk et al., 2019) and IPV (Fite et al., 2008; Gage, 2016; Goncy, 2020; Kimber et al., 2018; Murshid & Murshid, 2018; Smith et al., 2011; Stith et al., 2000), two on the heterogenetic continuity of parent-child aggression (G1) and IPV perpetration (G2) (Goncy et al., 2021; Li et al., 2020). This CMO is best explained by social learning theory, which posits that violence is used as an habitual response to conflict because of learned behaviors within the family where violent behavior modeled by adults provides scripts of violent behavior and teaches that violence is an acceptable mode to resolve problems (Bandura, 1977).

For example, Fite et al. (2008) used data from the longitudinal Child Development Project in the United States of 585 children recruited when entering reception class and their caregivers and found higher risk for aggression toward romantic partners in children of parents with violent relationships. They found that children whose parents have high levels of relationship conflict are modeled more aggressive and fewer nonaggressive responses to relationship conflict and therefore are more aggressive toward provocative situations in their own relationships. This is because they did not learn that a less aggressive response could achieve a similar or better outcome (Fite et al., 2008). Similarly, in a cross-sectional study of Haitian adolescents, the authors observed that in a context where violence and gender inequality are normalized at home, individuals learn to engage in interpersonal violence through observation, imitation, and modelling of significant others (Gage, 2016).

A global meta-analysis involving 98 unique samples found a small to moderate global effect for homotypical intergenerational transmission of IPV perpetration (Goncy, 2020). The authors used social learning theory to explain how individuals learn and replicate behaviors from their family of origin in their own relationships (Bandura, 1977). “Perhaps witnessing multiple forms of aggression, including physical forms such as hitting, as well as psychological forms, such as yelling or controlling, sends a powerful message that aggression is acceptable and normative in close relationships” (Goncy, 2020, p. 20). Although studies included in this review came from different contexts, little information was available about the contextual factors, for example, gender norms endorsed in the contexts studied, other than that domestic abuse in the parent generation occurred, and normalization of violence and harmful gender norms may have also been a mechanism.

Another global meta-analysis of 87 effect sizes found a small global effect for the intergenerational transmission of child maltreatment (G1) and IPV perpetration (G2), which was moderated by gender and stronger for men (S. Li et al., 2020). The authors explain this using the General Aggression Model (Anderson & Bushman, 2002), which posits that those with child maltreatment experience normalize the utility of violence and “form aggressive scripts” that they use to deal with stressors and frustrations in their intimate relationships, thereby resulting in the perpetration of IPV. This happens most commonly in the context of little family support and low resilience in the individual. Similarly, data from the Bangladesh Demographic and Health Survey 2007 of 3,396 men showed a significant association between childhood exposure to marital violence and marital violence perpetration in G2 (Murshid & Murshid, 2018). The authors observed that witnessing parental violence in childhood provides a justification for marital violence, therefore normalizing it and allowing it to perpetuate across generations, in line with social learning theory (Bandura, 1977). In the Netherlands, in a multi-generational qualitative study of eight families involved in violent crime, using archival data, criminal investigation files, and interviews with the police, social workers and probation offers, Spapens and Moors (2020) found that children learn that violence is an “acceptable strategy to dissolve disputes.” By observing their parents using violence in their personal conflicts, they understand that violence is efficient in producing a desired result through intimidation (Spapens & Moors, 2020).

In their analysis of the Rochester Youth Development Study and the Rochester Intergenerational Study, Augustyn et al. (2017) analyzed data from 371 parent–child dyads in which G3 children were at least 17 years old. They found that gang membership in fathers increases the likelihood of gang membership in their sons, moderated by the extent of father-son contact in late childhood with the highest risk of son’s involvement in gangs for those who see their fathers for at least an hour per week and those who live with their fathers (Augustyn et al., 2017). The relationship between parental and son gang membership is mediated by a father’s maltreatment of his son, which accounts for nearly 40% of the total effect of intergenerational gang membership. For mothers and daughters, there was a direct effect of gang membership in G2 on gang membership in G3. There was no evidence of intergenerational continuity of gang membership for father—daughter and mother-son dyads. Following Bandura’s social learning theory (Bandura, 1977), the authors hypothesize that abusing one’s child may model that physical violence is acceptable to address conflict hence increasing a child’s risk for violence continuity. In addition, they suggest that child neglect promotes indirect transmission of violence as it models those traditional responsibilities of parenthood, that is, caring for a child, are not important.

In a meta-analysis of 63 effect sizes examining the association between growing up in a violent home and perpetrating IPV, the authors found that association effects were stronger for men. They hypothesize that children model the behavior of their same-sex parent; therefore, boys mimic their father’s behavior of inflicting violence while girls observe their mothers as victims (Stith et al., 2000).

#### CMO 3: Emotion Dysregulation

***If*** a person has experienced CM or IPV throughout their lifespan, but particularly in early childhood, (C; G1), ***then*** they develop poor emotion regulation (M; G1), which may result in them perpetrating violence against their children or partners (O; G2).

Nine included studies supported this CMO (Bozzay et al., 2020; Choi et al., 2023; Domoney & Trevillion, 2021; Greene et al., 2020; Kim et al., 2009; Li et al., 2021; Martinez, 2020; Narayan et al., 2013; Reyes et al., 2015), three for the homotypical transmission of child maltreatment (Greene et al., 2020) and IPV (H. K. Kim et al., 2009; Martinez, 2020) and six for the heterotypical transmission of CM/IPV victimization (G1) to CM perpetration of G2 (Choi et al., 2023; Domoney & Trevillion, 2021), CM/IPV victimization (G1) and IPV perpetration (G2) (Narayan et al., 2013; Reyes et al., 2015) and family violence (G1) to youth violence (G2) (Bozzay et al., 2020; Li et al., 2021).

For example, Choi et al., 2023 examined the risk of inflicting child abuse among women who have experienced child abuse or IPV in adulthood using the Korean National Survey of Domestic Violence including *n* = 1,122 married women with children under the age of 18. The authors argue that the high levels of violence experienced in the mother generation, and in particular the IPV victimization, are harmful to the mother–child relationship due to women survivor’s difficulties in regulating their own emotions, which thereby increases the risk for abuse against their children. This highlights the importance of mental health support for women who have been victims of family violence (Choi et al., 2023).

In their analysis of the Safe Dates randomized trial with 2,343 adolescents in the United States, Reyes et al. (2015) investigated whether the relationship between witnessing interparental violence or experiencing child maltreatment and subsequent dating aggression was mediated by anger dysregulation or depression (Reyes et al., 2015). They found that anger dysregulation mediated the relationship between child maltreatment experience and dating aggression, but there was no evidence for an indirect effect of witnessing interparental violence through anger dysregulation on dating aggression. This is consistent with a body of literature that describes how violence experiences can interfere with emotional development and in particular emotion regulation (Jouriles et al., 2012). An inability to control emotions in relation to conflict can therefore increase the risk of dating aggression (Reyes et al., 2015). Depression was not a mediator for witnessing interparental violence nor experiencing child maltreatment on dating aggression in this study. Evidence on depression as a mediator between childhood violence experience and dating aggression is mixed. This supports the hypothesis that depression is more closely related to internalizing behaviors such as self-injurious behavior, rather than externalizing behaviors, such as dating aggression. Such a relationship could be gendered with men suffering from internalizing problems more likely to be violent toward women, and both men and women suffering from externalizing behavior more likely to engage in violence (Ellis et al., 2009; Yu et al., 2019).

Additionally, timing of experience of parental IPV seems to be critical in children’s expectations of social relationships and their ability to develop control of their negative behaviors and emotions as found in a longitudinal study of 168 low-income families in the United States. Experience of parental IPV in early childhood substantially increases the risk for dating violence in early adolescence, more so than the continuity or persistence of parental IPV experience through early and middle childhood (Narayan et al., 2013). The authors explain that this is because children are first learning how to regulate their emotions and control their behaviors in infancy and toddlerhood and thus have fewer positive experiences to guide how emotion regulation and interactions in relationships should look like.

Bozzay et al. (2020) investigated how personality traits may help explain relationships between witnessing family violence or experiencing child abuse and youth violent aggression in a sample of caregivers and adolescents (*n* = 237) in the United States. They found that witnessing family violence was linked with poorer impulse control and negative emotionality, defined as stress reaction and alienation, which increased levels of violent behavior. They also found that the experience of child abuse was linked with negative emotionality, which increased levels of violent behavior (Bozzay et al., 2020). The authors argue that children’s cognitive control processes are impacted by chronic and salient stressors such as family violence and that personality traits may be influenced by genes and their interaction with the environment. Similar findings were discussed by other authors who argued that witnessing parental IPV causes emotional distress, in the form of post-traumatic stress, anxiety, anger, and sadness, which then results in increased risk for violence perpetration (Martinez, 2020) and youth violence perpetration (Li et al., 2021).

In their analyses of data from the Oregon Youth Study and the Oregon Youth Couples Study, Kim et al. (2009) investigated the underlying mechanisms from parental emotion dysregulation via parental IPV to son’s IPV perpetration among 190 young men. They found that parental emotion dysregulation increased IPV, parental poor discipline and the son’s emotion dysregulation thereby increasing the son’s risk for IPV. There was an indirect effect of parent’s emotion dysregulation to son’s relationship conflict via son’s emotion dysregulation, and an indirect effect of parent’s emotion dysregulation to son’s emotion dysregulation through poor parental discipline (Kim et al., 2009). The authors therefore argue that emotion dysregulation is a major mechanism underlying the intergenerational transmission of relationship conflict. This is because people with poor emotion regulation rely on inappropriate strategies when with their partners and may also have poor self-regulatory abilities, which increase their risk for relationship conflict. As this study shows, emotion dysregulation may impair parenting and lead to inconsistent discipline practices, which further increase the risk of offspring emotion dysregulation (Kim et al., 2009).

#### CMO 4: High Vulnerability

***If*** parents experience a multitude of risk factors such as unemployment, illicit substance use, alcohol use, and mental health problems, they are more likely to engage in violent behaviors (C; G1), ***then*** successive generations may also experience these risk factors (M; G2) resulting in these children perpetrating violence (O, G2).

This CMO was supported by four studies (Besemer et al., 2017; Frisell et al., 2011; Jaffee et al., 2013; van Dijk et al., 2019). Three focused on the homotypical continuation of violent crime (Besemer et al., 2017; Frisell et al., 2011; van Dijk et al., 2019) and one on the continuation of child maltreatment/IPV (G1) and subsequent child maltreatment (G2) (Jaffee et al., 2013). High vulnerability is best explained through the risk factor model where many risk factors relating to family dysfunction for example, unemployment within the child’s environment, poverty, household instability at the structural level intersect and co-occur with psychosocial risk factors (e.g., substance use, poor parental mental health) and exacerbate each other creating an intergenerational vulnerability which can manifest in intergenerational violence continuity (Farrington, 2011).

For example, in a qualitative in-depth analysis of police, justice department, and child welfare files of 25 organized crime offenders in Amsterdam and their children, van Dijk et al. (2019) found intergenerational transmission of violent crime to be particularly pertinent from father to son. The authors highlighted that intergenerational continuity is a problem in high-crime families due to multiple mechanisms, one of which is where men with criminal backgrounds found families with women of similar backgrounds, also called “assortive mating” (Farrington, 2011). As the father is largely absent from the family due to being in prison or his involvement in criminal activity, the mother is tasked as the sole primary caregiver in the family. However, due to her own involvement in criminal activity and experience of instability and stress throughout her development, a stable home environment is not provided to the children by father or mother. This instability is then passed on, compromising positive development outcomes for the next generation (van Dijk et al., 2019) in line with resilience theory (Fergus & Zimmerman, 2005).

In their analysis of convictions for violent crime among 12.5 million individuals covering 32 years in the Swedish Multi-Generation Register, Frisell et al. (2011) found strong evidence for intergenerational transmission from parent to child with an odds of 3.5 and equally strong for other first-degree relatives, and somewhat lower odds for second-and third- degree relatives (Frisell et al., 2011). Interestingly, children who were given up for adoption also had increased odds for violent offending OR 1.9, as did children who were adopted into the family OR 1.5. This suggests that shared environment plays an important part in intergenerational violence transmission, but that there must also be a genetic effect. The odds for intergenerational transmission of violent offending in this study were much higher for women, and particularly high for mother–daughter pairs OR 6.3 and grandmother–grand-daughter pairs OR 2.0. Families in high socio-economic strata had higher odds of intergenerational violence transmission OR 5.8, explained by the fact that violent crime more commonly occurs in lower socio-economic strata, hence, shared familial risk factors are particularly salient and family ties more important to influence criminal behaviour in higher socio-economic strata. The authors suggest that the gender effects are explained by female-specific environmental (e.g., anti-social behavior by the mother) and female-specific genetic effects. This is supported by a high degree of assortive mating found in this study (Farrington, 2002), with convicted violent offenders more likely to marry women who were convicted violent offenders OR 5.2 (Frisell et al., 2011).

In their meta-analysis of 23 studies investigating the intergenerational transmission of violent behavior, Besemer et al. (2017) engage with the theory of the criminogenic environment (Farrington, 2011). This stipulates that people who offend are more likely to experience a multitude of risks in other areas of their lives such as poverty, substance use, antisocial features, and ill-health while living in an unstable home environment. Their children may therefore “be entrapped in poverty, have disrupted family lives, may experience single and teenage parenting, and may live in the most deprived neighborhoods” (Farrington, 2010) (p. 205). Criminal behavior is therefore not directly transmitted across generations but through a “continuity of a constellation of antisocial and criminogenic features” (Besemer et al., 2017) (p. 163). Besemer et al. (2017) also found that the intergenerational transmission is stronger from mothers to their offspring compared to fathers. As most caregiving tasks traditionally have been carried out by mothers, maternal imprisonment is more disruptive for children because children are more likely to move to live with other relatives leading to instability in their lives (Besemer et al., 2017).

Jaffee et al. (2013) in their analysis of 1116 parent–child dyads from the Environmental Risk Twin Study (E-Risk) in the UK found that mothers who had experienced childhood maltreatment were distinguished from those who had not by several factors. These factors included socioeconomic disadvantage, poor mental health, poor partner’s mental health, domestic partner violence, and low social support. These same factors also applied to their children who were physically maltreated. Intergenerational violence cycle maintainers reported higher levels of substance abuse, depression, social disadvantage, antisocial behavior, and domestic partner violence (Jaffee et al., 2013).

#### CMO 5: Impaired Relationships

***If*** parents experienced IPV or child maltreatment (C; G1), ***then*** they struggle to form safe and nurturing relationships with others including their partners and children (M; G2) resulting in intergenerational transmission of violence perpetration (O, G2).

Six studies investigated impaired relationships as underlying mechanisms for intergenerational transmission of violence perpetration (Adshead & Bluglass, 2001; Augustyn et al., 2017; Chapple, 2003; Jaffee et al., 2013; Schofield et al., 2013; Thornberry et al., 2013). All studies focused on the homotypical transmission of IPV, child maltreatment, or gang membership.

Schofield et al. (2013) conducted a meta-analysis of five studies focused on the intergenerational transmission of maltreatment and potential moderators. They investigated the role of safe, stable, and nurturing relationships in line with interactional theory, which posits that those who have experienced or engaged in violence will struggle forming positive relationships in adulthood (Thornberry et al., 2013). They found a strong association between child maltreatment in G1 and subsequent maltreatment of G2. However, they then went on to identify safe, stable, and nurturing relationships as protective moderators on the intergenerational continuity of maltreatment despite different measures and conceptualizations of safe, stable, and nurturing relationships in the included studies. For example, in their analysis of 1,116 families in the Environmental Risk (E-Risk) Longitudinal Twin Study in the United Kingdom, Jaffee et al. (2013) found substantial continuity of abuse from G1 to G2. The odds for children of mildly and severely maltreated mothers to experience maltreatment were 3.55 times and 5.31 times those of non-maltreated mothers. For families that maintained the intergenerational cycle of violence, mothers reported fewer warm relationships with their children and less trusting relationships with their partners.

Similarly, in their study of 980 grade 9 to 11 students in Arkansas, Chapple (2003) investigated social bonding/social control theory among boys and girls which states that inherently all of humanity has natural urges to act in aggressive and selfish ways and only attachment and bonds formed with pro-social values, people and institutions prevents acts of violence and aggression (Hirschi, 1969). The study found that adolescents who had witnessed parental violence reported lower mean levels of parental attachment and monitoring and higher mean levels of dating violence, and this was particularly true for girls. The study also found that parental attachment did not moderate the relationship between witnessing interparental violence and dating violence. Parental monitoring was however significantly associated with a reduction in violent offending in G2. Chapple argues that strong parental attachment and monitoring discourage dating violence perpetration, while weak parental monitoring and poor attachment are underlying mechanisms for intergenerational violence transmission of violence and aggression perpetration. This was however disputed in another study, which found that attachment and inconsistent discipline did not act as mediators of intergenerational gang membership between fathers and sons nor mothers and daughters, although mothers involved in gangs were more likely to parent their daughters inconsistently (Augustyn et al., 2017).

## Discussion

In recent years, an increasing number of studies have focused on the strengths of the association of different types of intra-familial violence perpetration across generations. These studies have established that there is a small to moderate overall effect for the intergenerational transmission of IPV, child abuse, and violent crime (Besemer et al., 2017; Goncy, 2020; Kimber et al., 2018; Stith et al., 2000). However, simply knowing that a link exists is not sufficient for targeted interventions. It is crucial to understand why this link exists and what factors contribute to the intergenerational transmission of violence perpetration. The purpose of this realist review was therefore to increase our understanding of the underlying mechanisms of this intergenerational transmission of violence perpetration and examine if existing theories are supported by our final problem theories.

The realist review drew on 28 studies and identified five CMOs on how violence perpetration is transmitted from one generation to the next, namely, normalization of violence and harmful gender norms, modeling and imitation, emotion dysregulation, high vulnerability, and impaired relationships. Some of these are focused within a context, where violence occurs within the family or outside of the family, or where violence is not condoned by some members of society, and where an underlying mechanism is the adoption of harmful gender norms. We found that in these societal contexts characterized by high levels of violence, normalization and modeling are important mechanisms for homo- and heterotypical transmission of violence across generations predominantly driven by harmful gender norms, irrespective of the country setting. Normalization and modeling are interlinked phenomena, where normalization of violence and harmful gender norms occur at the macro societal level; while modeling and imitation occur at the micro family level. As violence has been systematically used as a tool of oppression and gender norms operate in all contexts, other forms of violence that occur at the country level for example, coup d’états can increase endorsement of harmful gender norms at the societal and family level. Where children witness and experience violence, they learn that the use of violence is a normal and acceptable tool to address and solve problems and can therefore be legitimately used in conflict. Violence might also be seen as a normal and expected part of normative development to fit in with one’s peer group. Children who witness and experience violence learn fewer non-aggressive responses to conflict and imitate their parent’s behaviors in conflict settings. They may also learn that to stay safe, one must be violent, and that meeting violence with violence ensures personal protection.

These CMOs therefore support existing overlapping theories such as social learning theory (Bandura, 1977), social network theory (Tracy et al., 2016), power theory (Straus et al., 1980), and the general aggression model (Anderson & Bushman, 2002). Where this acceptance of family violence is compounded by a societal context characterized by inequitable gender norms and norms which condone violence, this can provide further support for the normalization of violence and harmful gender norms, and support and create expectation for subscription to the use of violence as a part of culture (Nisbett, 2003). Normalization and modeling are important mechanisms for targeted intervention such as those set out in the World Health Organization’s INSPIRE framework focused on addressing gender norms and beliefs at the societal level and creating positive role models (World Health Organization, 2016), which aim to prevent violence against children but have not been evaluated or implemented with a view to preventing intergenerational violence transmission. Evidence-based interventions that could target normalization and modeling by teaching healthy interpersonal interactions are needed from as early as primary school.

Emotion dysregulation was another important mechanism for hetero- and homotypical intergenerational violence transmission. Any type of violence experience increases risk for depression, anxiety, and post-traumatic stress (Miliauskas et al., 2022; White et al., 2024) and can also reduce impulse and anger control (Neilson et al., 2023), particularly where experience of violence is persistent and occurs in early childhood. Following the bio-developmental framework, one reason for this can be that these early life stressors affect neurobiological systems and brain development and functioning, which reduce the body’s ability to cope with stressful experiences, that is, by developing mental disorders or perpetrating violence (Shonkoff, 2010). The emotion dysregulation mechanism also supports the stress process model based on social stress theory by which violence experience is considered a source of stress, which is linked to poor mental health, which is a mediator of stress to a manifestation of stress, that is, violence perpetration (Pearlin et al., 1981). Similarly, strain theory describes violence perpetration as a coping mechanism to deal with persistent adversities related to crime and violence over which individuals have no control (Agnew, 2007), and is as such linked to poor emotional health. Emotion regulation is a skillset that is modeled by close relationships throughout childhood, and children who grow up with adversity often do not learn these emotion regulation skills. Emotion regulation could be an important focus for targeted interventions, particularly with mounting evidence on the effectiveness of interventions to improve mental health together with parenting practices (Bütikofer et al., 2024; Cluver et al., 2018; Gervinskaitė-Paulaitienė et al., 2023; Mooren et al., 2023; Narayan et al., 2021), thereby improving outcomes on multiple sustainable development goals at the same time for both parents and children.

High vulnerability characterized by multiple shared risk factors which co-occur, intersect, and exacerbate each other were also identified as an important underlying mechanism for hetero- and homotypical intergenerational violence transmission. These could occur across the socio-ecological environments a person interacts with include substance use, poverty, teenage parenthood, or anti-social behavior, amongst others. This is in line with socio-ecological systems theory (Bronfenbrenner, 1979), later updated to person-process-context-time (Bronfenbrenner & Morris, 2007; Navarro et al., 2022), which has some overlap with the theory around criminogenic environments (Farrington, 2011). Both theories posit that it is not perpetration per se that is transmitted across generations, but that a whole host of risk factors related to disadvantage in a person’s life interact with each other in the absence of protective or promotive factors over a person’s life course, thereby perpetuating disadvantage, and creating an environment where violence is transmitted from one generation to the next. One of the ways in which this disadvantage is perpetuated is through neglectful or absent parenting coupled with authoritarian discipline in line with the theory of assortive mating, where people with criminal records and similar disorganized parenting styles marry each other (Farrington, 2002). A lot of blame in the criminology literature is attributed toward mothers as not being able to provide stable, safe, and nurturing environments for their children while the children’s fathers are in prison, and this narrative needs to shift. Addressing disadvantage is important to prevent intergenerational transmission of violence. Rigorous evidence is available for comprehensive child and family welfare services with a focus on poverty alleviation and safe and stable housing (World Health Organization, 2016) showing reductions in violence against children and women. Future research needs to confirm whether these will also be effective in reducing intergenerational transmission.

Impaired relationships formed the final important mechanism of intergenerational violence transmission in this review with evidence solely supporting homotypical transmission of perpetration. In line with attachment theory (Bowlby, 1982), growing up in families where children experience violence as infants compromises their ability to form secure attachments, so insecure attachments are formed that promote distrust within relationships, with an inability to manage distress that can lead to responding to others with aggression. Social bonding and social control theory assume that these attachments, or pro-social bonds, help people to control their selfish and aggressive behavior and therefore prevent violence from occurring (Hirschi, 1969). When people have engaged in violent crime in adolescence, interactional theory posits that they will have problematic transitions into adult roles, which affects how they parent; that is, low involved parenting, inconsistent monitoring, and with harsh disciplinary methods, therefore perpetuating the cycle of violence perpetration (Thornberry, 2005). Interventions supporting people in forming safe, stable, and nurturing relationships are therefore important to address this underlying mechanism of intergenerational violence transmission. Parenting and IPV prevention interventions are robust evidence-based approaches to improve parenting and reduce child abuse, IPV, and violent offending (Farrington et al., 2022; Meinck et al., 2019; Morrison et al., 2014; WHO, 2022).

Our CMOs did not support a number of theories that are commonly used in intergenerational violence transmission research. For example, a bio-developmental framework (Shonkoff, 2010) is a plausible theory to explain emotion dysregulation, due to biological changes within the body, however, none of the included studies provided empirical evidence for these changes across generations and their causal impact or association with violence perpetration. Social information processing was also not supported by any of the included studies (Dodge et al., 1990). Social bonding theory was poorly supported; our data found that strong pro-social bonds do prevent intergenerational transmission of violence, however, there was no evidence to support the theory that every person is born with a drive to act aggressively (Hirschi, 1969). Genetic theory focused on inheritance of genes related to impulsivity and self-control was supported by one study only, though it showed strong effects for intergenerational transmission of violent crime even in children who were adopted outside the family (Frisell et al., 2011).

### Strengths and Limitations

This realist review used a systematic and transparent search and synthesis strategy and followed a pre-registered protocol. Studies were double reviewed and double extracted, and CMOs were developed through regular team discussions. CMOs were supported by the data from included studies and through existing theories on intergenerational violence perpetration. Limitations include the dearth of data available in the existing literature on the intergenerational transmission of violence perpetration of types of violence beyond child maltreatment, IPV, and violent crime, and the distinct lack of data from low-and middle-income countries up to the end search date in December 2021. Data were strongest for intra-familial violence types, and no studies focused on intergenerational transmission of peer violence perpetration or community violence perpetration. For many studies, it was unclear who had inflicted child maltreatment or who was the perpetrator of IPV witnessed, nor was there a lot of information on the specific subtypes of child maltreatment (physical, emotional, sexual, or neglect) or IPV (physical, emotional, sexual, financial, coercive control) experiences witnessed or perpetrated. Many studies also focused their discussion almost exclusively on the statistical associations they had uncovered in analysis. They did not elaborate on what the underlying mechanisms for their findings might be nor used theoretical frameworks to explain their findings.

This review has identified a number of gaps that need to be addressed through future research. For instance, the field needs to move beyond testing for the existence of intergenerational transmission toward testing theories of violence perpetration within and across generations. Large multi-generational administrative or survey data sets could help in adjudicating and developing new theories for intergenerational violence transmission. Further research is also needed on identifying who the victims/perpetrators are in generation 1 and in which situations they experience or inflict violence and in building the evidence around intergenerational transmission with a specific focus on the role of gender. Future studies should allow look at subtypes of child maltreatment and IPV and other types of violence outside of the family for example, peer or community violence in building the evidence around intergenerational transmission of violence. Most importantly, intervention research needs to move beyond the focus on single-generation violence prevention and examine long-term effects of interventions on violence perpetration across multiple generations.

**Table:** Implications for Practice, Policy, and Research.

*Implications for practice* • Parenting interventions, which also include emotion regulation components, are indicated and should be widely available but particularly for parents who have experienced violence-related trauma • Safe and stable housing combined with harm reduction and poverty alleviation are important mechanisms to provide stable homes for children growing up in adversity to stop the cycle of violence • Interventions that transform gender norms and power relations and provide safety for survivors of violence are important focal points for evidence-based interventions
*Implications for policy* Primary prevention of violence is indicated to prevent the cycle of violence transmission through: • Universally accessible provision of welfare services including parenting interventions, cash benefits, housing, and harm reduction are indicated to prevent intergenerational violence transmission • Clear and visible campaigns and messaging of those in the public space rejecting harmful gender norms and violence in any form
*Implications for research* • More research is needed focusing on the intergenerational transmission of other types of violence that occur outside the home environment and on the linkages between these other types of violence and family violence • More information is needed on whether different subtypes of child maltreatment or IPV have different underlying mechanisms of violence transmission • Investigations into the role of gender and who perpetrates violence in G1 are needed as well as the situational contexts in which violence occurs • Intervention research and funders should focus on long-term follow-ups to investigate intervention effects over longer periods of time and multiple generations.

## Conclusion

The cycle of intergenerational violence perpetration is harmful and must be stopped. Understanding the contexts and underlying mechanisms under which violence is transmitted from one generation is helpful to identify potential mechanisms for intervention. Findings from this review suggest five clear mechanisms, which can be addressed by targeted interventions. Addressing harmful and violence-endorsing norms and beliefs, improving mental health, providing welfare and social services that address social determinants of health, and improving parent–child relationships are supported by evidence in providing a service framework that can interrupt the intergenerational cycle of violence perpetration.

## Supplemental Material

sj-docx-1-tva-10.1177_15248380251361468 – Supplemental material for What Are the Mechanisms Underpinning Intergenerational Transmission of Violence Perpetration? A Realist Review Supplemental material, sj-docx-1-tva-10.1177_15248380251361468 for What Are the Mechanisms Underpinning Intergenerational Transmission of Violence Perpetration? A Realist Review by Franziska Meinck, Mengyao Lu, Deeksha Suresh, Melis Cetin, Lakshmi Neelakantan, Chad Hemady, Hannabeth Franchino-Olsen, Nataly Woollett, GJ Melendez-Torres, Andrea Gonzalez and Nicola Christofides in Trauma, Violence, & Abuse
