# Supplementary material for: What Are the Mechanisms Underpinning Intergenerational Transmission of Violence Perpetration? A Realist Review
Source: Trauma Violence Abuse. 2025 Sep 1;27(4):1798–815. doi: 10.1177/15248380251361468 (PMC13578666; doi:10.1177/15248380251361468)
Supplement: Supplementary file 1 [file sj-docx-1-tva-10.1177_15248380251361468.docx]

**Supplementary material**

Contents

[**Supplement 1:** Theories of intergenerational violence perpetration	2](#_Toc176776559)

[**Supplement 2 :** Search terms for stage 1	9](#_Toc176776560)

[**Supplement 3:** Search terms for stage 2	11](#_Toc176776561)

[**Supplement 4:** Search terms for stage 3	19](#_Toc176776562)

[**Supplement 5:** Included studies	21](#_Toc176776563)

# **Supplement 1:** Theories of intergenerational violence perpetration

| Theory name | Theory explanation |
| --- | --- |
| Assortive mating | Men with a criminal record marry women with criminal records. Children in these families often experience neglect, lack of supervision and authoritarian discipline, all of which increase the risk for children to engage in criminal violent behaviour(D. Farrington, 2002). |
| Attachment Theory | Attachment, or the early bond formed between infants and their primary caregiver are thought to serve as the foundational basis of children’s orientation in the world and shaping subsequent interpersonal interactions and relationships (Bowlby, 1982). Secure attachment develops through consistent sensitive and responsive caregiving to the infant’s cues (i.e., crying). Attachment theory suggests that when this security is disrupted, and in extreme cases, such as child maltreatment, children come to view the world and others (internal working models) with hostility and respond with aggression, which can turn into violent behaviour in adulthood (Egeland, 1993). Early childhood experiences of violence provide the child with an internal working model (or understanding of relationships) that is seen as unpredictable and dangerous. Attachment to parents, or other caregivers, during this critical life stage can be an important protective factor against violent behaviour. |
| Bio-developmental framework | Gene-environment interactions in the early years of life can have lasting effects on the neurobiological system of a child. Environmental stressors, such as witnessing domestic abuse in utero and early childhood can affect brain development, brain functioning, the metabolic system and immune response, all of which impact one’s ability to cope with stressful experience later in life (Shonkoff, 2010). The ongoing effect of early adversity mediates the relationship between adverse experiences and continuation of violence in later life (Kimber et al., 2018). |
| Biological/Genetic Theory | There has been one study to indicate that there has been intergenerational transmission of violence may be heritable (Frisell et al., 2011) however, it is unknown how biological and social factors interact to produce violent behaviour in offspring. Violence is particularly relevant within a biopsychosocial framework because of its association with hereditary and socially modelled factors such as impulsivity and low self-control. Genes of interest include genes related to neuronal signalling (dopamine and serotonin signalling, neurotransmitters that influence mental health and behaviour) such as 5-HTTLPR, Catechol-O-methyltransferase, MAOA, and dopamine D4 receptor, which have all been shown to increase antisocial behaviour and impulsive violence (Iofrida et al., 2014). Future work should focus on how witnessing parental violence (or exposure to violence in childhood), calibrates biological systems, which may have already been vulnerable to express violent behaviour. |
| Cultural cognitive theory | Nisbett posits that people from different cultures have different fundamental beliefs about the world and differ also in how they perceive and reason about the world (Nisbett, 2003). Children who grow up socialised into a system that endorses violence, then experience the use of violence as part of their culture which is transmitted through caregiver-child interactions. These children then use violence as a primary means to resolve conflict and respond with violence to stimuli because of the ‘system of thought’ they have grown into (Kim, 2012). |
| Developmental Traumatology Model | Increased risk for posttraumatic stress disorder is a direct consequence of violence experiences. PTSD is defined by avoidance, hyperarousal and mistrust of others therefore, when left untreated, make it more likely for affected parents to commit acts of omission (failure to protect a child/neglect) and more difficult to learn effective parenting strategies or feel joy from being a parent thereby resulting in abuse (De Bellis, 2001). |
| General Aggression Model | The General Aggression Model is an integrative approach that draws on social learning theory, script theory and social interaction theory amongst others and considers risk factors for aggression within the social, cognitive, personality developmental and biological make up of a person (Allen et al., 2018). It is a broad framework for understanding aggression. The model posits that these risk factors interact with each other, particularly on the personal and situational level. Intergenerational violence can occur because a person who believes that violence is normal and useful due to being exposed to violence in their upbringing is more likely to themselves use violence, particularly if they are in a situation that has also been shown to be a risk factor for violence e.g., aggressive and loud environment. These situational and personal risks affect a person’s appraisal and cognition processes and these then influence their violent or non-violent behaviour. |
| Interactional theory | This is an extension of the Interactional Theory of Offending which posits that people who have engaged in criminal behaviours as an adolescent have problems transitioning into adult roles including being a parent which affects the quality of their parenting. While parents who engage in antisocial norms serve as models for anti-social behaviours for their children directly, predominantly an indirect pathway via family conflict, hostility and low quality of parenting increases offspring risk for violent offending. Parenting in these families is often characterised by low affective ties, little involvement with offspring, inconsistent monitoring and explosive physical discipline (Thornberry, 2005). |
| Power theory | Power theory positions the roots of violence in both culture and the family structure (Straus et al., 1980). Normative attitudes and social acceptance of violence, gender inequality and family conflict all interact and shape intimate partner violence, in addition to increasing tensions within the family. These tensions can arise due to economic hardships, power imbalances between partners and high levels of stress. Like social learning theory, it is posited that children learn to use violence to address conflict through witnessing abuse or experiencing it themselves (Straus et al., 1980) . |
| Resiliency Theory | Resiliency theory posits that there are contextual, social and individual promotive factors that interrupt pathways from adverse experiences to risk behaviours and poor health and mental health outcomes, such as violence perpetration. These promotive factors are categorised into factors at the level of the individual such as self-esteem (assets) and factors in the environment of the individual such as supportive adults, green neighbourhoods, progressive gender norms, or skill building opportunities (resources) (Fergus & Zimmerman, 2005). In the absence of these promotive factors, intergenerational violence continues. |
| Risk factors and mechanisms | Intergenerational continuity of delinquent behaviour is not directly transmitted from one generation to the next but through a ‘larger cycle of deprivation and antisocial behaviour’(Farrington, 2011) (p. 132). Shared risk factors which are associated with an increased risk for violent behaviour include poverty, substance abuse, teen pregnancy and parental conflict. Growing up in these criminogenic environments support the continuity of violence perpetration by providing a continuity of a constellation of antisocial features. |
| Social bonding and social control | Hirschi posits that every person is born with a “hedonistic drive to act in the kinds of selfish and aggressive ways that lead to criminal behaviour” (Hirschi, 1969). People who are able to control their urges to act violently towards others have formed bonds to prosocial people, institutions and values. He defines these as attachment, commitment, involvement and belief; together these prosocial bonds control people’s behaviour and help them adhere to social conventions and laws. According to Hirschi, people who engage in violent behaviour across generations lack these prosocial bonds. However, some research has shown that men who had violent family histories and then developed strong attachments with significant others were less likely to be violent towards their female partners than those men who had not formed significant attachments (Lackey & Williams, 1995). |
| Social Information Processing | Social information processing models posit that severe physical abuse early in childhood creates deficiencies in ability to process social information such that children interpret ambiguous or harmless interactions as hostile and react with aggression (Dodge et al., 1990). |
| Social Learning | Through social learning processes such as observational learning and modelling, violence is used as a habitual response to conflict with intimate partners through channels of learned behaviour (Bandura, 1979). Because the family is the main socializing influence and the main source of child learning, aggression modelled between parents not only provides scripts for violent behaviours but also teaches the child what is acceptable behaviour and models of problem solving. Children may learn that violence is an effective means of conflict resolution with intimate partners or a means of gaining control. Essentially violence is normalised and legitimised. It has also been hypothesised that violence continues in adulthood if it has been reinforced by positive outcomes or has served a purpose – thus modelled behaviour is more likely to be adopted if it is perceived to result in advantageous outcomes with few negative consequences. Finally, children in violent households may not have the opportunity to learn positive consequences of socially acceptable methods for resolving interpersonal communication and conflict such as negotiation, verbal reasoning, self-calming tactics and active listening (Foshee et al., 1999). Moreover, according to Sutherland’s Differential Association Theory, people will commit delinquent acts when they have learned more motivations to break rather than to follow the law(Sutherland & Cressey, 1955). When parents are more frequent offenders, children have more opportunities to observe and imitate their parents’ delinquent behaviour and motivations and thus one would expect more offspring crime. |
| Social Network | Transmission of violence through interpersonal networks via exposure to violence and experiences of individual victimization or perpetration (Tracy et al., 2016). Being in high-risk environments with shared violent experiences and norms may also increase the risk of further victimization by accepting violence as a norm, predisposing one to engage in violence in defence or retaliation, and sharing violent experiences with others in their network (Tracy et al., 2016). |
| Socio-ecological theory | Exposure to violence and early adversity may be part of overarching multi-systemic environmental interactions and create contexts for children that perpetuate generational transmission of aggression (Bronfenbrenner, 1979). The ecological-translational model takes into account the interaction of multiple factors across various contexts (Cicchetti & Lynch, 1993). It is hypothesised that each level contains risk and protective factors for the individual which increase or reduce the risk and may moderate outcomes associated with child abuse (Zielinski & Bradshaw, 2006). The updated Process, Person, Context, Time (PPCT) model takes into account synergistic and interrelated influences of proximal processes, person characteristics and time on development but has not been applied to intergenerational violence transmission(Bronfenbrenner & Morris, 2007; Navarro et al., 2022). |
| Strain Theory | Strain theory suggests that persistent, negative experiences that are unjust, high in magnitude, associated with low social control, and model violence and crime lead youth to engage in violence and crime to cope(R. Agnew, 2001; R. S. Agnew, 2007). These strain experiences include but are not limited to child abuse, parental rejection, negative school experiences, and abusive peer relations, although the latter has been understudied. |
| Stress Process model | The Stress Process model is based on social stress theory and posits that “the process of social stress can be seen as combining three major conceptual domains: the sources of stress, the mediators of stress, and the manifestations of stress” (Pearlin et al., 1981). Experiences such as child maltreatment are seen as stressful life events and are linked to poor physical and mental health outcomes in later life which can manifest behaviourally, such as through maltreatment of one’s own child. These pathways are mediated by positive and negative events and processes (e.g., coping) which increase or decrease the risk for continuity of violence. |

**References**

Agnew, R. (2001). Building on the Foundation of General Strain Theory: Specifying the Types of Strain Most Likely to Lead to Crime and Delinquency. *Journal of Research in Crime and Delinquency*, *38*(4). https://journals.sagepub.com/doi/10.1177/0022427801038004001

Agnew, R. S. (2007). Strain theory and violent behavior. In *The Cambridge handbook of violent behavior and aggression* (pp. 519–529). Cambridge University Press. https://doi.org/10.1017/CBO9780511816840.027

Allen, J. J., Anderson, C. A., & Bushman, B. J. (2018). The General Aggression Model. *Current Opinion in Psychology*, *19*, 75–80. https://doi.org/10.1016/j.copsyc.2017.03.034

Bandura, A. (1979). The social learning perspective: Mechanisms of aggression. In *Psychology of crime and criminal justice* (pp. 198–236). Waveland Press.

Bowlby, J. (1982). *Attachment and loss* (2nd ed). Basic Books.

Bronfenbrenner, U. (1979). *The ecology of human development: Experiments by nature and design*. Harvard University Press.

Bronfenbrenner, U., & Morris, P. A. (2007). The Bioecological Model of Human Development. In *Handbook of Child Psychology*. John Wiley & Sons, Inc. https://doi.org/10.1002/9780470147658.chpsy0114

Cicchetti, D., & Lynch, M. (1993). Toward an ecological/transactional model of community violence and child maltreatment: Consequences for child development. *Psychiatry*, *56*, 96–118.

De Bellis, M. (2001). Developmental traumatology: The psychobiological development of maltreated children and its implications for research, treatment and policy. *Development and Psychopathology*, *13*, 539–564.

Dodge, K., Bates, J., & Pettit, G. (1990). Mechanisms in the cycle of violence. *Science*, *250*(4988), 1678–1683.

Egeland, B. (1993). A History of Abuse is a Major Risk Factor for Abusing the Next Generation. In R. J. Gelles & D. R. Loseke (Eds.), *Current Controversies on Family Violence* (pp. 197–208). SAGE Publications.

Farrington, D. (2002). Developmental criminology and risk-focused prevention. In *The Oxford Handbook of Criminology* (pp. 657–701). Oxford University Press.

Farrington, D. P. (2011). Families and crime. In *Crime and public policy* (pp. 130–157). Oxford University Press.

Fergus, S., & Zimmerman, M. A. (2005). ADOLESCENT RESILIENCE: A Framework for Understanding Healthy Development in the Face of Risk. *Annual Review of Public Health*, *26*(Volume 26, 2005), 399–419. https://doi.org/10.1146/annurev.publhealth.26.021304.144357

Foshee, V. A., Bauman, K. E., & Linder, G. F. (1999). Family Violence and the Perpetration of Adolescent Dating Violence: Examining Social Learning and Social Control Processes. *Journal of Marriage and Family*, *61*(2), 331–342. https://doi.org/10.2307/353752

Frisell, T., Lichtenstein, P., & Långström, N. (2011). Violent crime runs in families: A total population study of 12.5 million individuals. *Psychological Medicine*, *41*(1), 97–105. https://doi.org/10.1017/S0033291710000462

Hirschi, T. (1969). *Causes of Delinquency*. University of California Press.

Iofrida, C., Palumbo, S., & Pellegrini, S. (2014). Molecular genetics and antisocial behavior: Where do we stand? *Experimental Biology and Medicine (Maywood, N.J.)*, *239*(11), 1514–1523. https://doi.org/10.1177/1535370214529508

Kim, K. (2012). The role of culture in theories of the intergenerational transmission of violence. *Child & Family Social Work*, *17*(4), 395–405. https://doi.org/10.1111/j.1365-2206.2011.00793.x

Kimber, M., Adham, S., Gill, S., McTavish, J., & MacMillan, H. L. (2018). The association between child exposure to intimate partner violence (IPV) and perpetration of IPV in adulthood—A systematic review. *Child Abuse & Neglect*, *76*, 273–286. https://doi.org/10.1016/j.chiabu.2017.11.007

Lackey, C., & Williams, K. R. (1995). Social Bonding and the Cessation of Partner Violence Across Generations. *Journal of Marriage and Family*, *57*(2), 295–305. https://doi.org/10.2307/353684

Navarro, J. L., Stephens, C., Rodrigues, B. C., Walker, I. A., Cook, O., O’Toole, L., Hayes, N., & Tudge, J. R. H. (2022). Bored of the rings: Methodological and analytic approaches to operationalizing Bronfenbrenner’s PPCT model in research practice. *Journal of Family Theory & Review*, *14*(2), 233–253. https://doi.org/10.1111/jftr.12459

Nisbett, R. E. (2003). *The geography of thought: How Asians and Westerners think differently ... and why* (pp. xxiii, 263). Free Press.

Pearlin, L. I., Menaghan, E. G., Lieberman, M. A., & Mullan, J. T. (1981). The Stress Process. *Journal of Health and Social Behavior*, *22*(4), 337–356. https://doi.org/10.2307/2136676

Shonkoff, J. P. (2010). Building a New Biodevelopmental Framework to Guide the Future of Early Childhood Policy. *Child Development*, *81*(1), 357–367. https://doi.org/10.1111/j.1467-8624.2009.01399.x

Straus, M., Gelles, R., & Steinmetz, S. (1980). *Behind Closed Doors: Violence in the American Family* (1st ed.). Anchor Press. https://www.routledge.com/Behind-Closed-Doors-Violence-in-the-American-Family/Straus-Gelles-Steinmetz/p/book/9781412805919

Sutherland, E. H., & Cressey, D. R. (1955). *Principles of Criminology*. Lippincott.

Thornberry, T. P. (2005). Explaining Multiple Patterns of Offending across the Life Course and across Generations. *The ANNALS of the American Academy of Political and Social Science*, *602*(1), 156–195. https://doi.org/10.1177/0002716205280641

Tracy, M., Braga, A. A., & Papachristos, A. V. (2016). The Transmission of Gun and Other Weapon-Involved Violence Within Social Networks. *Epidemiologic Reviews*, *38*(1), 70–86. https://doi.org/10.1093/epirev/mxv009

Zielinski, D. S., & Bradshaw, C. P. (2006). Ecological Influences on the Sequelae of Child Maltreatment: A Review of the Literature. *Child Maltreatment*, *11*(1), 49–62. https://doi.org/10.1177/1077559505283591

# **Supplement 2 :** Search terms for stage 1

| database | search term | result |
| --- | --- | --- |
| embase | ((child or children or infant* or baby or babies or teen* or adolescen* or youth* or toddler* or young person or young people or minor or teen*) and (maltreat* abuse or punish* or mistreat* or neglect* or abandon* or harm* or offen* or assault* or rap* or molest* or exploit* or spank* or hit* or smack or victimi* or violence) and (intergeneration* or multigeneration* or transgeneration* or generation*) and (transmission or pathway* or continuities) and (review* or overview* or meta-analy* or metaanaly* or metanaly* or meta-regression* or metaregression* or meta NEAR 1 regression* or synthes* or metasynthes* or meta-synthes* or metaethnograph* or meta-ethnograph*)).ti,ab. limit 3 to yr="1990 -Current" | 320 |
| global health |  |  |
| medline |  |  |
| psycinfo |  |  |
| pubmed | (((child[Title/Abstract] OR children[Title/Abstract] OR infant*[Title/Abstract] OR baby[Title/Abstract] OR babies[Title/Abstract] OR teen*[Title/Abstract] OR adolescen*[Title/Abstract] OR youth*[Title/Abstract] OR toddler*[Title/Abstract] OR young person[Title/Abstract] OR young people[Title/Abstract] OR minor[Title/Abstract] OR teen*[Title/Abstract]) AND (maltreat* abuse[Title/Abstract] OR punish*[Title/Abstract] OR mistreat*[Title/Abstract] OR neglect*[Title/Abstract] OR abandon*[Title/Abstract] OR harm*[Title/Abstract] OR offen*[Title/Abstract] OR assault*[Title/Abstract] OR rap*[Title/Abstract] OR molest*[Title/Abstract] OR exploit*[Title/Abstract] OR spank*[Title/Abstract] OR hit*[Title/Abstract] OR smack[Title/Abstract] OR victimi*[Title/Abstract] OR violence[Title/Abstract])) AND (intergeneration*[Title/Abstract] OR multigeneration*[Title/Abstract] OR transgeneration*[Title/Abstract] OR generation*[Title/Abstract])) AND (review*[Title/Abstract] OR overview*[Title/Abstract] OR meta-analy*[Title/Abstract] OR metaanaly*[Title/Abstract] OR metanaly*[Title/Abstract] OR meta-regression*[Title/Abstract] OR metaregression*[Title/Abstract] OR meta NEAR 1 regression*[Title/Abstract] OR synthes*[Title/Abstract] OR metasynthes*[Title/Abstract] OR meta-synthes*[Title/Abstract] OR metaethnograph*[Title/Abstract] OR meta-ethnograph*[Title/Abstract]) | 336 |
| sociological abstract | ab(child OR children OR infant* OR baby OR babies OR teen* OR adolescen* OR youth* OR toddler* OR young person OR young people OR minor OR teen*) AND ab(maltreat* abuse OR punish* OR mistreat* OR neglect* OR abandon* OR harm* OR offen* OR assault* OR rap* OR molest* OR exploit* OR spank* OR hit* OR smack OR victimi* OR violence) AND ab(intergeneration* OR multigeneration* OR transgeneration* OR generation*) AND ab(transmission OR pathway* OR continuities) AND ab((review* OR overview* OR meta-analy* OR metaanaly* OR metanaly* OR meta-regression* OR metaregression* OR (meta NEAR/1 regression*) OR synthes* OR metasynthes* OR meta-synthes* OR metaethnograph* OR meta-ethnograph*))Publication date from 1990/01/01 to 2021/12/31 | 74 |
| proquest |  |  |
| cochrane | child or children or infant* or baby or babies or teen* or adolescen* or youth* or toddler* or young person or young people or minor or teen* in Title Abstract Keyword AND maltreat* abuse or punish* or mistreat* or neglect* or abandon* or harm* or offen* or assault* or rap* or molest* or exploit* or spank* or hit* or smack or victimi* or violence in Title Abstract Keyword AND intergeneration* or multigeneration* or transgeneration* or generation* in Title Abstract Keyword AND transmission or pathway* or continuities in Title Abstract Keyword AND review* OR overview* OR meta-analy* OR metaanaly* OR metanaly* OR meta-regression* OR metaregression* OR (meta NEAR/1 regression*) OR synthes* OR metasynthes* OR meta-synthes* OR metaethnograph* OR meta-ethnograph* in Title Abstract Keyword - with Cochrane Library publication date Between Jan 1990 and May 2021 (Word variations have been searched) | 58 |
| total |  | 778 |
| total after deduplicated | | 590 |

# **Supplement 3:** Search terms for stage 2

| **database** | **search terms** | **results** | **Note** |
| --- | --- | --- | --- |
| Embase 1974 to present, Database Field Guide Global Health 1973 to 2021 Week 43, Database Field Guide Medline (Ovid MEDLINE® Epub Ahead of Print, In-Process & Other Non-Indexed Citations, Ovid MEDLINE® Daily and Ovid MEDLINE®) 1946 to present, Database Field Guide PsycINFO 1806 to present | ((child or children or infant* or baby or babies or teen* or adolescen* or youth* or toddler* or young person or young people or minor or teen* or son or daughter or offspring or descendant) and (maltreat* or abuse or punish* or mistreat* or neglect* or abandon* or harm* or offen* or assault* or rap* or molest* or exploit* or spank* or hit* or smack or victimi* or violence or war* or conflict* or intimate partner violence or criminal behavio* or witness* violence or intergeneration* trauma or harsh parenting or abusive parenting) and (intergeneration* or multigeneration* or transgeneration* or generation* or transmission or pathway* or continuities or mechanism* or perpetrat*) and (qualitative or interview or focus group or ethnography or grounded theory or narrative research or case stud* or lived experience* or life experience*)).ab. limit 1 to yr="1990 - 2021" | 6837 | exported |
| PubMed | (((child[Title/Abstract] OR children[Title/Abstract] OR infant*[Title/Abstract] OR baby[Title/Abstract] OR babies[Title/Abstract] OR teen*[Title/Abstract] OR adolescen*[Title/Abstract] OR youth*[Title/Abstract] OR toddler*[Title/Abstract] OR young person[Title/Abstract] OR young people[Title/Abstract] OR minor[Title/Abstract] OR teen*[Title/Abstract] OR son[Title/Abstract] OR daughter[Title/Abstract] OR offspring[Title/Abstract] OR descendant[Title/Abstract]) AND (maltreat*[Title/Abstract] OR abuse[Title/Abstract] OR punish*[Title/Abstract] OR mistreat*[Title/Abstract] OR neglect*[Title/Abstract] OR abandon*[Title/Abstract] OR harm*[Title/Abstract] OR offen*[Title/Abstract] OR assault*[Title/Abstract] OR rap*[Title/Abstract] OR molest*[Title/Abstract] OR exploit*[Title/Abstract] OR spank*[Title/Abstract] OR hit*[Title/Abstract] OR smack[Title/Abstract] OR victimi*[Title/Abstract] OR violence[Title/Abstract] OR war*[Title/Abstract] OR conflict*[Title/Abstract] OR intimate partner violence[Title/Abstract] OR criminal behavio*[Title/Abstract] OR witness* violence[Title/Abstract] OR intergeneration* trauma[Title/Abstract] OR harsh parenting[Title/Abstract] OR abusive parenting[Title/Abstract])) AND (intergeneration*[Title/Abstract] OR multigeneration*[Title/Abstract] OR transgeneration*[Title/Abstract] OR generation*[Title/Abstract] OR transmission[Title/Abstract] OR pathway*[Title/Abstract] OR continuities[Title/Abstract] OR mechanism*[Title/Abstract] OR perpetrat*[Title/Abstract])) AND (qualitative[Title/Abstract] OR interview[Title/Abstract] OR focus group[Title/Abstract] OR ethnography[Title/Abstract] OR grounded theory[Title/Abstract] OR narrative research[Title/Abstract] OR case stud*[Title/Abstract] OR lived experience*[Title/Abstract] OR life experience*[Title/Abstract]) Filters: from 1990 - 2021 | 1571 | exported |
| Proquest thesis | ab(child OR children OR infant* OR baby OR babies OR teen* OR adolescen* OR youth* OR toddler* OR young person OR young people OR minor OR teen* OR son OR daughter OR offspring OR descendant) AND ab(maltreat* OR abuse OR punish* OR mistreat* OR neglect* OR abandon* OR harm* OR offen* OR assault* OR rap* OR molest* OR exploit* OR spank* OR hit* OR smack OR victimi* OR violence OR war* OR conflict* OR intimate partner violence OR criminal behavio* OR witness* violence OR intergeneration* trauma OR harsh parenting OR abusive parenting) AND ab(intergeneration* OR multigeneration* OR transgeneration* OR generation* OR transmission OR pathway* OR continuities OR mechanism* OR perpetrat*) AND ab(qualitative OR interview OR focus group OR ethnography OR grounded theory OR narrative research OR case stud* OR lived experience* OR life experience*) | 5314 | exported |
| sociological abstract | ab(child OR children OR infant* OR baby OR babies OR teen* OR adolescen* OR youth* OR toddler* OR young person OR young people OR minor OR teen* OR son OR daughter OR offspring OR descendant) AND ab(maltreat* OR abuse OR punish* OR mistreat* OR neglect* OR abandon* OR harm* OR offen* OR assault* OR rap* OR molest* OR exploit* OR spank* OR hit* OR smack OR victimi* OR violence OR war* OR conflict* OR intimate partner violence OR criminal behavio* OR witness* violence OR intergeneration* trauma OR harsh parenting OR abusive parenting) AND ab(intergeneration* OR multigeneration* OR transgeneration* OR generation* OR transmission OR pathway* OR continuities OR mechanism* OR perpetrat*) AND ab(qualitative OR interview OR focus group OR ethnography OR grounded theory OR narrative research OR case stud* OR lived experience* OR life experience*) Jan 1 1990 - Nov 1 2021 | 2597 | exported |
| Pubmed | ((((child[Title/Abstract] OR children[Title/Abstract] OR infant*[Title/Abstract] OR baby[Title/Abstract] OR babies[Title/Abstract] OR teen*[Title/Abstract] OR adolescen*[Title/Abstract] OR youth*[Title/Abstract] OR toddler*[Title/Abstract] OR young person[Title/Abstract] OR young people[Title/Abstract] OR minor[Title/Abstract] OR teen*[Title/Abstract] OR son[Title/Abstract] OR daughter[Title/Abstract] OR offspring[Title/Abstract] OR descendant[Title/Abstract]) AND (maltreat*[Title/Abstract] OR abuse[Title/Abstract] OR punish*[Title/Abstract] OR mistreat*[Title/Abstract] OR neglect*[Title/Abstract] OR abandon*[Title/Abstract] OR harm*[Title/Abstract] OR offen*[Title/Abstract] OR assault*[Title/Abstract] OR rap*[Title/Abstract] OR molest*[Title/Abstract] OR exploit*[Title/Abstract] OR spank*[Title/Abstract] OR hit*[Title/Abstract] OR smack[Title/Abstract] OR victimi*[Title/Abstract] OR violence[Title/Abstract] OR war*[Title/Abstract] OR conflict*[Title/Abstract] OR intimate partner violence[Title/Abstract] OR criminal behavio*[Title/Abstract] OR witness* violence[Title/Abstract] OR intergeneration* trauma[Title/Abstract] OR harsh parenting[Title/Abstract] OR abusive parenting[Title/Abstract])) AND (intergeneration*[Title/Abstract] OR multigeneration*[Title/Abstract] OR transgeneration*[Title/Abstract] OR generation*[Title/Abstract] OR transmission[Title/Abstract] OR pathway*[Title/Abstract] OR continuities[Title/Abstract] OR mechanism*[Title/Abstract] OR perpetrat*[Title/Abstract])) AND (post-traumatic growth[Title/Abstract] OR resilien*[Title/Abstract] OR protective factor*[Title/Abstract])) AND (qualitative[Title/Abstract] OR interview[Title/Abstract] OR focus group[Title/Abstract] OR ethnography[Title/Abstract] OR grounded theory[Title/Abstract] OR narrative research[Title/Abstract] OR case stud*[Title/Abstract] OR lived experience*[Title/Abstract] OR life experience*[Title/Abstract]) | 96 | protective factors |
| Embase 1974 to present, Database Field Guide Global Health 1973 to 2021 Week 43, Database Field Guide Medline (Ovid MEDLINE® Epub Ahead of Print, In-Process & Other Non-Indexed Citations, Ovid MEDLINE® Daily and Ovid MEDLINE®) 1946 to present, Database Field Guide PsycINFO 1806 to present | ((child or children or infant* or baby or babies or teen* or adolescen* or youth* or toddler* or young person or young people or minor or teen* or son or daughter or offspring or descendant) and (maltreat* or abuse or punish* or mistreat* or neglect* or abandon* or harm* or offen* or assault* or rap* or molest* or exploit* or spank* or hit* or smack or victimi* or violence or war* or conflict* or intimate partner violence or criminal behavio* or witness* violence or intergeneration* trauma or harsh parenting or abusive parenting) and (intergeneration* or multigeneration* or transgeneration* or generation* or transmission or pathway* or continuities or mechanism* or perpetrat*) and (post-traumatic growth or resilien* or protective factor*) and (qualitative or interview or focus group or ethnography or grounded theory or narrative research or case stud* or lived experience* or life experience*)).ab. | 412 | exported |
| proquest theses | ((child or children or infant* or baby or babies or teen* or adolescen* or youth* or toddler* or young person or young people or minor or teen* or son or daughter or offspring or descendant) and (maltreat* or abuse or punish* or mistreat* or neglect* or abandon* or harm* or offen* or assault* or rap* or molest* or exploit* or spank* or hit* or smack or victimi* or violence or war* or conflict* or intimate partner violence or criminal behavio* or witness* violence or intergeneration* trauma or harsh parenting or abusive parenting) and (intergeneration* or multigeneration* or transgeneration* or generation* or transmission or pathway* or continuities or mechanism* or perpetrat*) and (post-traumatic growth or resilien* or protective factor*) and (qualitative or interview or focus group or ethnography or grounded theory or narrative research or case stud* or lived experience* or life experience*)).ab. | 370 | exported |
| sociological abstract | same as above | 130 |  |
| Total |  | 17327 |  |

# **Supplement 4:** Search terms for stage 3

| **Database** | **Search terms** | **Results** |
| --- | --- | --- |
| Embase 1974 to present, Database Field Guide Global Health 1973 to 2021 Week 43, Database Field Guide Medline (Ovid MEDLINE® Epub Ahead of Print, In-Process & Other Non-Indexed Citations, Ovid MEDLINE® Daily and Ovid MEDLINE®) 1946 to present, Database Field Guide PsycINFO 1806 to present | (child OR children OR infant* OR baby OR babies OR teen* OR adolescen* OR youth* OR toddler* OR young person OR young people OR minor OR teen* OR son OR daughter OR offspring OR descendant) AND (vigilantism OR community violence OR youth violence OR gang violence OR bullying OR violent crime OR sexual exploitation OR dating violence OR sibling violence OR sibling aggression OR war OR conflict OR traffick*) AND (intergeneration* OR multigeneration* OR transgeneration* OR generation* OR transmission OR pathway* OR continuities OR mechanism* OR perpetrat* OR trajectory OR facilitat* OR inherenc* OR genetic*) | 911 |
| PubMed | (child OR children OR infant* OR baby OR babies OR teen* OR adolescen* OR youth* OR toddler* OR young person OR young people OR minor OR teen* OR son OR daughter OR offspring OR descendant) AND (vigilantism OR community violence OR youth violence OR gang violence OR bullying OR violent crime OR sexual exploitation OR dating violence OR sibling violence OR sibling aggression OR war OR conflict OR traffick*) AND (intergeneration* OR multigeneration* OR transgeneration* OR generation* OR transmission OR pathway* OR continuities OR mechanism* OR perpetrat* OR trajectory OR facilitat* OR inherenc* OR genetic*) | 6294 |
| Proquest thesis | (child OR children OR infant* OR baby OR babies OR teen* OR adolescen* OR youth* OR toddler* OR young person OR young people OR minor OR teen* OR son OR daughter OR offspring OR descendant) AND (vigilantism OR community violence OR youth violence OR gang violence OR bullying OR violent crime OR sexual exploitation OR dating violence OR sibling violence OR sibling aggression OR war OR conflict OR traffick*) AND (intergeneration* OR multigeneration* OR transgeneration* OR generation* OR transmission OR pathway* OR continuities OR mechanism* OR perpetrat* OR trajectory OR facilitat* OR inherenc* OR genetic*) | 77 |
| sociological abstract | (child OR children OR infant* OR baby OR babies OR teen* OR adolescen* OR youth* OR toddler* OR young person OR young people OR minor OR teen* OR son OR daughter OR offspring OR descendant) AND (vigilantism OR community violence OR youth violence OR gang violence OR bullying OR violent crime OR sexual exploitation OR dating violence OR sibling violence OR sibling aggression OR war OR conflict OR traffick*) AND (intergeneration* OR multigeneration* OR transgeneration* OR generation* OR transmission OR pathway* OR continuities OR mechanism* OR perpetrat* OR trajectory OR facilitat* OR inherenc* OR genetic*) | 235 |
| Total |  | 7517 |

# **Supplement 5:** Included studies ordered by CMO*

| CMOs |  | Study | Sample | Types of violence | Analysis results | Location |
| --- | --- | --- | --- | --- | --- | --- |
| Normalisation of violence and harmful gender norms |  | Domoney & Trevillion 2021 | Qualitative study with ten men with DV histories engaged in a peri-natal parenting program | Parental IPV (G1, witnessed by G2), or G1 childhood maltreatment against G2) – IPV perpetration (G2) | Men’s past experiences of violence shape their behaviours in relationships and as parents both in ways that may interrupt cycles of violence or may repeat cycles of violence. | United Kingdom |
| Normalisation of violence and harmful gender norms |  | Gage 2016 | 342 high school students (54% girls), cross-sectional baseline evaluation of SAFE Dates | parental spousal violence (G1, witnessed by G2), - physical, psychological and sexual dating violence perpetration (G2) | The relationship between father and mother perpetrated IPV and dating violence perpetration for girls was mediated by personal acceptance of dating violence. Boys who had witnessed their father perpetrating IPV were more likely to perpetrate psychological dating violence. Mother perpetrated IPV reduced risk for psychological, physical and sexual dating violence in boys. Peer and personal tolerance of dating violence were associated with dating violence perpetration. Boy’s witnessing of parental IPV and dating violence perpetration was moderated by perceived peer attitudes towards dating violence. | Haiti |
| Normalisation of violence and harmful gender norms |  | Kimber et al., 2018 | Systematic review of 19 studies | Parental IPV (G1, witnessed by G2) - IPV perpetration (G2) | Sixteen included studies found association between children’s exposure to parental IPV and IPV perpetration in adulthood. Studies predominantly focused on witnessing of physical IPV and perpetration of physical IPV in heterosexual families. In particular violence by men against women increased risk for IPV perpetration among sons and daughters. | global |
| Normalisation of violence and harmful gender norms |  | Li et al., 2021 | Meta-analysis of 87 effect sizes | Child maltreatment (G1)– IPV perpetration (G1) | Childhood maltreatment, specifically physical, emotional and sexual abuse) is associated with later IPV perpetration, particularly for men | global |
| Normalisation of violence and harmful gender norms |  | Martinez 2020 | Six Latino young adults (3 men, 3 women) aged 21 and 25 | Parental IPV (G1 witnessed by G2) – IPV perpetration (G2) | Young people who witnessed domestic abuse between their parents described significant emotional distress and has together with a normalisation of these behaviours in their context influenced what they view as appropriate in their relationships with normalisation and acceptance of violence | USA |
| Normalisation of violence and harmful gender norms |  | Reyes et al., 2015 | 2343 adolescents in Grade 8 and 9 (50% girls) participating in the randomised controlled trail of the Safe Dates intervention in rural public schools | Parental IPV (G1, witnessed by G2) / physical abuse experience against G2 – dating aggression (G2) | Experience of physical abuse predicted anger dysregulation which increased risk for dating aggression. Experience of physical abuse also increased harmful normative beliefs about dating violence which in turn increased risk for dating aggression. | USA |
| Normalisation |  | Stith, S et al., 2000 | Meta-analysis of 63 effect sizes from published and unpublished studies | Growing up in violent home (witnessing violence between G1/ experience of violence by G1 against G2 (G2) – perpetration of IPV (G2) | Men who grow up in violent homes are more likely to perpetrate IPV. Men were more likely to perpetrate IPV following the experience of child abuse or witnessing domestica abuse, as were people recruited through clinical settings. | Global |
| Modelling and imitation |  | Augustyn et al 2017 | 371 parent-child dyads in the Rochester Youth Development Study. Parents are 60% men, children are 51% men | Gang membership | Gang membership continuity happens between mothers and daughters and father and sons if father and son have regular contact and is mediated by experience of child abuse in father-son dyads. | USA |
| Modelling and imitation |  | Fite et al., 2008 | 484 adolescents and their fathers and mothers in the prospective longitudinal Child Development Project | parental violent relationships (G1) –aggression towards intimate partners (G2) | Parental conflict directly predicts offspring aggression towards intimate partners. This relationship is mediated by offspring response (aggressive, neutral or proactive) to a particular situation and how they might evaluate a particular situation and their response to it. No significant gender differences | USA |
| Modelling and imitation |  | Gage 2016 | 342 high school students (54% girls), cross-sectional baseline evaluation of SAFE Dates | parental spousal violence (G1, witnessed by G2), - physical, psychological and sexual dating violence perpetration (G2) | The relationship between father and mother perpetrated IPV and dating violence perpetration for girls was mediated by personal acceptance of dating violence. Boys who had witnessed their father perpetrating IPV were more likely to perpetrate psychological dating violence. Mother perpetrated IPV reduced risk for psychological, physical and sexual dating violence in boys. Peer and personal tolerance of dating violence were associated with dating violence perpetration. Boy’s witnessing of parental IPV and dating violence perpetration was moderated by perceived peer attitudes towards dating violence. | Haiti |
| Modelling and imitation |  | Goncy 2020 | Meta-analysis of 94 samples (66 peer-reviewed journal articles or dissertations) | Parent- child aggression (G1) and dating aggression (G2) | Small global effect (r=.155) for association between parent-child aggression and dating aggression was found. Effect sizes were slightly larger for psychological dating aggression compared to physical dating aggression. | Global |
| Normalisation of violence and harmful gender norms |  | Goncy et al., 2021 | Meta-analysis of 98 samples (70 peer-reviewed journal articles or dissertations) | Parental IPV (G1, witnessed by G2) – dating aggression (G2) | Small global effect (r=.162) for association between parental IPV and dating aggression perpetration. Effect of witnessing physical and emotional parental IPV was more strongly associated with dating aggression than witnessing just psychological parental IPV. | Global |
| Modelling and imitation |  | Kimber et al., 2018 | Systematic review of 19 studies | Parental IPV (G1, witnessed by G2) - IPV perpetration (G2) | Sixteen included studies found association between children’s exposure to parental IPV and IPV perpetration in adulthood. Studies predominantly focused on witnessing of physical IPV and perpetration of physical IPV in heterosexual families. In particular violence by men against women increased risk for IPV perpetration among sons and daughters. | global |
| Modelling and imitation |  | Li et al., 2020 | Meta-analysis of 87 effect sizes | Child maltreatment (G1)– IPV perpetration (G1) | Childhood maltreatment, specifically physical, emotional and sexual abuse) is associated with later IPV perpetration, particularly for men | global |
| Modelling and imitation |  | Murshid & Murshid 2018 | 3396 men who had been married in the Demographic and Health Survey 2007 aged 15-54 | Parental IPV (G1, witnessed by G2) – IPV perpetration (G2) | Witnessing parental domestic abuse (aOR 2.45) and endorsing marital violence (aOR 2.21) increases odds of perpetrating violence | Bangladesh |
|  |  |  |  |  |  |  |
| Modelling and imitation |  | Smith et al., 2011 | Rochester Youth Development Study of 1000 youth followed from age 14 to adulthood. 73% men and 85% African American or Hispanic and their caregivers | Parental IPV (G1)/child abuse against G2 – IPV perpetration (G2) | Adolescents who witnessed parental domestic abuse had increased risk of perpetrating IPV aged 21-23. This relationship was mediated by adolescent dating violence in early adulthood. Child abuse experience was a significant direct predictor of adult IPV perpetration | USA |
| Modelling and imitation |  | Spapens & Moors 2020 | Multi-generational qualitative study using archival data of 7 families with a leader of a criminal organisation in one generation | Violent criminal behaviour | Most family members across the three generations have criminal records but only 2 have had further crime leaders. In these families, partners and friends are selected from similar circles, predominantly those who already have a criminal record. | Netherlands |
| Modelling and imitation |  | Stith, S et al., 2000 | Meta-analysis of 63 effect sizes from published and unpublished studies | Growing up in violent home (witnessing violence between G1/ experience of violence by G1 against G2 (G2) – perpetration of IPV (G2) | Men who grow up in violent homes are more likely to perpetrate IPV. Men were more likely to perpetrate IPV following the experience of child abuse or witnessing domestica abuse, as were people recruited through clinical settings. | Global |
| Modelling and imitation |  | Van Dijk et al., 2019 | Qualitative in-depth analysis of archival data (police, child protection, criminal justice files) of 25 members of criminal organisations and their 45 children | Violent criminal behaviours | Sons of individuals involved in organised crime also get involved in organised crime, whereas daughters do not, and if they do, they only get involved in minor crime. Lack of parenting skills, father’s reputation and modelling of behaviour increase risk for intergenerational continuity. Maternal parenting skills may be protective, as is involvement for child protective services for daughters. | Netherlands |
| Emotion dysregulation |  | Bozzay et al., 2020 | 237 adolescents (57% girls) with and without mental health treatment histories, cross-sectional | Family violence (G1, witnessed by G2)/ G1 child maltreatment against G2 – violent aggression (G2) | The relationship between witnessing family violence and violent aggression is fully mediated by personality trait. The relationship between child maltreatment and violent aggression is partially mediated by personality traits. Results hold for both boys and girls. | USA |
| Emotion dysregulation |  | Choi et al., 2023 | 1122 married women who participated in the 2010 National Survey of Domestic Violence | CM experience (G1)/ IPV victimization (G1) – CM perpetration G1 against G2 | Child abuse in G1 directly predicts child abuse perpetration by G1 against G2. This relationship is partially mediated by maternal IPV victimization. Being in a current loving relationship moderated the pathway from child abuse victimization to child abuse perpetration. | Korea |
| Emotion dysregulation |  | Domoney & Trevillion 2021 | Qualitative study with ten men with DV histories engaged in a peri-natal parenting program | Parental IPV (G1, witnessed by G2), or G1 childhood maltreatment against G2) – IPV perpetration (G2) | Men’s past experiences of violence shape their behaviours in relationships and as parents both in ways that may interrupt cycles of violence or may repeat cycles of violence. | United Kingdom |
| Emotion dysregulation |  | Greene et al., 2020 | Systematic review of 97 studies | Child maltreatment (G1) – violent parenting practices against G2 | Parents who have witnessed domestic abuse or experienced physical abuse in childhood are more likely to inflict child abuse or neglect with greatest risk for those parents with multiple abusive experiences in childhood. Mental health and women’s IPV experience is a mediator of these relationships. Little evidence for link between childhood sexual and infliction of child abuse or neglect. | global |
| Emotion dysregulation |  | Kim et al., 2009 | Longitudinal data across 21 years of 190 men and their mothers and fathers. Oregon study | Parental IPV (G1) – IPV perpetration (G2) | The relationship between parental IPV and offspring IPV perpetration was mediated by emotion dysregulation and poor parenting skills. Parental poor emotion regulation was associated with offspring poor emotion regulation and increased IPV perpetration. Parents who had poor emotion dysregulation were more likely to have poor discipline skills which increased offspring risk for emotion dysregulation and IPV perpetration. | USA |
| Emotion dysregulation |  | Li et al., 2021 | 1100 adolescents, (50% girls) in a two-wave longitudinal study | Parental IPV (G1 witnessed by G2) or physical abuse by G1 against G2 – youth violence (G2) | Family violence predicts youth violence, however the relationship is mediated by normative beliefs approving of violence and negative emotions such as depression, anxiety, hostility, and paranoid ideation.. | China |
| Emotion dysregulation |  | Martinez 2020 | Six Latino young adults (3 men, 3 women) aged 21 and 25 | Parental IPV (G1 witnessed by G2) – IPV perpetration (G2) | Young people who witnessed domestic abuse between their parents described significant emotional distress and has together with a normalisation of these behaviours in their context influenced what they view as appropriate in their relationships with normalisation and acceptance of violence | USA |
| Emotion dysregulation |  | Narayan et al., 2013 | 168 (87 men, 81 women) low-income families from Minnesota Longitudinal Study of Risk and Adaptation and their mothers | Parental IPV (G1)/ child maltreatment by G1 against G2 – IPV perpetration (G2) | Witnessing parental domestic abuse in early and middle childhood was directly associated with IPV perpetration at age 23. Witnessing parental IPV also was associated with experience of child maltreatment which predicted externalising behaviour, which predicted life stress at 23 years and IPV perpetration at 23 years. | USA |
| Emotion dysregulation |  | Reyes et al., 2015 | 2343 adolescents in Grade 8 and 9 (50% girls) participating in the randomised controlled trail of the Safe Dates intervention in rural public schools | Parental IPV (G1, witnessed by G2) / physical abuse experience against G2 – dating aggression (G2) | Experience of physical abuse predicted anger dysregulation which increased risk for dating aggression. Experience of physical abuse also increased harmful normative beliefs about dating violence which in turn increased risk for dating aggression. | USA |
| High Vulnerability |  | Besemer 2012 | Meta-analysis of 23 studies in 25 publications including mothers and fathers | Violent criminal behaviours | Children with parents engaging in criminal violent behaviours have 2.4 times the odds of engaging in criminal behaviours themselves. Strongest transmission was from mother to daughter and to son and then fathers to daughters and sons. | global |
| High Vulnerability |  | Friselll et al., 2011 | 12.5 million individuals in the Swedish Multi-Generation Register, 11,878,407 parent-child dyads | Violent crime (G1) – violent crime (G2) | Parent-child transmission of violent crime OR 3.5 (3.5-3.6) with higher odds for female-female dyads OR 6.4 (5.7-6.9) | Sweden |
| High Vulnerability |  | Jaffee et al., 2013 | 1116 mother-child dyads from the Environmental Risk (E-Risk) Longitudinal Twin Study | Childhood maltreatment (G1)/ IPV experience (G1) – child maltreatment (G2) | Children whose mothers had experienced maltreatment had 3.5 times the odds of also experiencing maltreatment. Maternal IPV victimisation was the sole risk factor for intergenerational continuity of maltreatment compared to cycle breakers. Maternal warmth and a health relationship between mothers and their partners reduced risk for intergenerational continuity | United Kingdom |
| High Vulnerability |  | Van Dijk et al., 2019 | Qualitative in-depth analysis of archival data (police, child protection, criminal justice files) of 25 members of criminal organisations and their 45 children | Violent criminal behaviours | Sons of individuals involved in organised crime also get involved in organised crime, whereas daughters do not, and if they do, they only get involved in minor crime. Lack of parenting skills, father’s reputation and modelling of behaviour increase risk for intergenerational continuity. Maternal parenting skills may be protective, as is involvement for child protective services for daughters. | Netherlands |
| Impaired relationships |  | Adshead & Bluglass 2001 | Three-generational case history (grandmother, mother & daughter) | CM (G1) – Factitious illness by Proxy (G2) against G3 | Childhood abuse and neglect and frequent moves in childhood may create insecure attachments which are continued from one generation to the next and increase the risk for child maltreatment through factitious illness by proxy | United Kingdom |
| Impaired relationships |  | Augustyn et al 2017 | 371 parent-child dyads in the Rochester Youth Development Study. Parents are 60% men, children are 51% men | Gang membership | Gang membership continuity happens between mothers and daughters and father and sons if father and son have regular contact and is mediated by experience of child abuse in father-son dyads. | USA |
| Impaired relationships |  | Chapple 2003 | 980 grade nine to eleven students, 51% girls, cross-sectional | Parental violence (G1, witnessed by G2) – dating violence offending (G2) | Witnessing interparental violence is associated with increased risk for dating violence offending, as is low parental monitoring but this relationship is not moderated by parental attachment. | USA |
| Impaired relationships |  | Jaffee et al., 2013 | 1116 mother-child dyads from the Environmental Risk (E-Risk) Longitudinal Twin Study | Childhood maltreatment (G1)/ IPV experience (G1) – child maltreatment (G2) | Children whose mothers had experienced maltreatment had 3.5 times the odds of also experiencing maltreatment. Maternal IPV victimisation was the sole risk factor for intergenerational continuity of maltreatment compared to cycle breakers. Maternal warmth and a health relationship between mothers and their partners reduced risk for intergenerational continuity | United Kingdom |
| Impaired relationships |  | Schofield et al., 2013 | Meta-analysis of 5 studies using prospective longitudinal data | Child maltreatment (G1)-use of child maltreatment against G2 | There is intergenerational continuity of child maltreatment but safe, stable and nurturing relationships have a protective moderating effect on intergenerational continuity | UK and USA |
| Impaired relationships |  | Thornberry et al., 2013 | Rochester Youth Development study age 14-31 (30% girls) | Child maltreatment (G1) – child maltreatment against G2 by G1 | Parents who experienced child maltreatment have 2.6 times the odds to perpetrate child maltreatment. Healthy adult romantic relationships and adult-child relationships (good attachment, parental satisfaction) were protective of intergenerational continuity of maltreatment. | USA |
|  |  |  |  |  |  |  |

***Note:** Some studies appear multiple times as they covered multiple CMOs

**References**

Adshead, G., & Bluglass, K. (2001). A vicious circle: Transgenerational attachment representations in a case of factitious illness by proxy. *Attachment & Human Development*, *3*(1), 77–95. https://doi.org/10.1080/14616730010024780

Augustyn, M. B., Ward, J. T., & Krohn, M. D. (2017). Exploring Intergenerational Continuity in Gang Membership. *Journal of Crime and Justice*, *40*(3), 252–274. https://doi.org/10.1080/0735648X.2017.1337556

Besemer, S. (2012). Specialized Versus Versatile Intergenerational Transmission of Violence: A New Approach to Studying Intergenerational Transmission from Violent Versus Non-Violent Fathers: Latent Class Analysis. *Journal of Quantitative Criminology*, *28*(2), 245–263. https://doi.org/10.1007/s10940-011-9141-y

Bozzay, M., Lendi, J., & Verona, E. (2020). Family Violence Pathways and Externalizing Behavior in Youth. *Journal of Interpersonal Violence*, *35*(23–24), 5726–5752.

Chapple, C. L. (2003). Examining intergenerational violence: Violent role modeling or weak parental controls? *Violence and Victims*, *18*(2), 143–162. https://doi.org/10.1891/vivi.2003.18.2.143

Choi, S., Kim, J. Y., Kim, E., & Oh, S. (2023). Intergenerational Transmission of Child Abuse Among Married Women in South Korea: A Multigroup Analysis of Empathetic Communication (TSL: Thanks, Sorry, and Love). *Journal of Interpersonal Violence*, *38*(11–12), 7315–7334. https://doi.org/10.1177/08862605221145719

Domoney, J., & Trevillion, K. (2021). Breaking the cycle of intergenerational abuse: A qualitative interview study of men participating in a perinatal program to reduce violence. *Infant Mental Health Journal*, *42*(2), 206–221. https://doi.org/10.1002/imhj.21886

Fite, J. E., Bates, J. E., Holtzworth-Munroe, A., Dodge, K. A., Nay, S. Y., & Pettit, G. S. (2008). Social Information Processing Mediates the Intergenerational Transmission of Aggressiveness in Romantic Relationships. *Journal of Family Psychology*, *22*(3), 367–376. https://doi.org/10.1037/0893-3200.22.3.367

Frisell, T., Lichtenstein, P., & Långström, N. (2011). Violent crime runs in families: A total population study of 12.5 million individuals. *Psychological Medicine*, *41*(1), 97–105. https://doi.org/10.1017/S0033291710000462

Gage, A. J. (2016). Exposure to Spousal Violence in the Family, Attitudes and Dating Violence Perpetration Among High School Students in Port-au-Prince. *Journal of Interpersonal Violence*, *31*(14), 2445–2474. https://doi.org/10.1177/0886260515576971

Goncy, E. A. (2020). A meta-analysis of interparental aggression with adolescent and young adult physical and psychological dating aggression. *Psychology of Violence*, *10*(2), 212–222. https://doi.org/10.1037/vio0000266

Goncy, E., Basting, E., & Dunn, C. (2021). A Meta-Analysis Linking Parent-to-Child Aggression and Dating Abuse During Adolescence and Young Adulthood. *Trauma, Violence & Abuse*, *22*(5), 1248–1261.

Greene, C. A., Haisley, L., Wallace, C., & Ford, J. D. (2020). Intergenerational effects of childhood maltreatment: A systematic review of the parenting practices of adult survivors of childhood abuse, neglect, and violence. *Clinical Psychology Review*, *80*, 101891. https://doi.org/10.1016/j.cpr.2020.101891

Jaffee, S. R., Bowes, L., Ouellet-Morin, I., Fisher, H. L., Moffitt, T. E., Merrick, M. T., & Arseneault, L. (2013). Safe, stable, nurturing relationships break the intergenerational cycle of abuse: A prospective nationally representative cohort of children in the United Kingdom. *The Journal of Adolescent Health: Official Publication of the Society for Adolescent Medicine*, *53*(4 Suppl), S4-10. https://doi.org/10.1016/j.jadohealth.2013.04.007

Kim, H. K., Pears, K. C., Capaldi, D. M., & Owen, L. D. (2009). Emotion Dysregulation in the Intergenerational Transmission of Romantic Relationship Conflict. *Journal of Family Psychology : JFP : Journal of the Division of Family Psychology of the American Psychological Association (Division 43)*, *23*(4), 585–595. https://doi.org/10.1037/a0015935

Kimber, M., Adham, S., Gill, S., McTavish, J., & MacMillan, H. L. (2018). The association between child exposure to intimate partner violence (IPV) and perpetration of IPV in adulthood—A systematic review. *Child Abuse & Neglect*, *76*, 273–286. https://doi.org/10.1016/j.chiabu.2017.11.007

Li, S. D., Xiong, R., Liang, M., Zhang, X., & Tang, W. (2021). Pathways From Family Violence to Adolescent Violence: Examining the Mediating Mechanisms. *Frontiers in Psychology*, *12*. https://www.frontiersin.org/articles/10.3389/fpsyg.2021.611006

Li, S., Zhao, F., & Yu, G. (2020). A meta-analysis of childhood maltreatment and intimate partner violence perpetration. *Aggression and Violent Behavior*, *50*, 101362. https://doi.org/10.1016/j.avb.2019.101362

Martinez, A. M. (2020). *Domestic Violence Among Latinos and its Effect on the View of Marital Relationships in Young Adults Who Witnessed it as Children*. The Chicago School of Professional Psychology.

Murshid, N. S., & Murshid, N. (2018). Intergenerational Transmission of Marital Violence: Results From a Nationally Representative Sample of Men. *Journal of Interpersonal Violence*, *33*(2), 211–227. https://doi.org/10.1177/0886260515604413

Narayan, A. J., Englund, M. M., & Egeland, B. (2013). Developmental timing and continuity of exposure to interparental violence and externalizing behavior as prospective predictors of dating violence. *Development and Psychopathology*, *25*(4 Pt 1), 973–990. https://doi.org/10.1017/S095457941300031X

Reyes, H. L. M., Foshee, V. A., Fortson, B. L., Valle, L. A., Breiding, M. J., & Merrick, M. T. (2015). Longitudinal Mediators of Relations Between Family Violence and Adolescent Dating Aggression Perpetration. *Journal of Marriage and the Family*, *77*(7), 1016–1030. https://doi.org/10.1111/jomf.12200

Schofield, T. J., Lee, R. D., & Merrick, M. T. (2013). Safe, stable, nurturing relationships as a moderator of intergenerational continuity of child maltreatment: A meta-analysis. *The Journal of Adolescent Health: Official Publication of the Society for Adolescent Medicine*, *53*(4 Suppl), S32-38. https://doi.org/10.1016/j.jadohealth.2013.05.004

Smith, C. A., Ireland, T. O., Park, A., Elwyn, L., & Thornberry, T. P. (2011). Intergenerational continuities and discontinuities in intimate partner violence: A two-generational prospective study. *Journal of Interpersonal Violence*, *26*(18), 3720–3752. https://doi.org/10.1177/0886260511403751

Spapens, T., & Moors, H. (2020). Intergenerational transmission and organised crime. A study of seven families in the south of the Netherlands. *Trends in Organized Crime*, *23*(3), 227–241. https://doi.org/10.1007/s12117-019-09363-w

Stith, S. M., Rosen, K. H., Middleton, K. A., Busch, A. L., Lundeberg, K., & Carlton, R. P. (2000). The Intergenerational Transmission of Spouse Abuse: A Meta-Analysis. *Journal of Marriage and Family*, *62*(3), 640–654. https://doi.org/10.1111/j.1741-3737.2000.00640.x

Thornberry, T. P., Henry, K. L., Smith, C., Ireland, T. O., Greenman, S. J., & Lee, R. D. (2013). Breaking the Cycle of Maltreatment: The Role of Safe, Stable, and Nurturing Relationships. *The Journal of Adolescent Health : Official Publication of the Society for Adolescent Medicine*, *53*(4 0), S25–S31. https://doi.org/10.1016/j.jadohealth.2013.04.019

van Dijk, M., Kleemans, E., & Eichelsheim, V. (2019). Children of Organized Crime Offenders: Like Father, Like Child? An Explorative and Qualitative Study Into Mechanisms of Intergenerational (Dis)Continuity in Organized Crime Families. *European Journal on Criminal Policy and Research*, *25*(4), 345–363. https://doi.org/10.1007/s10610-018-9381-6
